# Multicenter Evaluation of the Accelerate PhenoTest BC Kit for Rapid Identification and Phenotypic Antimicrobial Susceptibility Testing Using Morphokinetic Cellular Analysis

**DOI:** 10.1128/JCM.01329-17

**Published:** 2018-03-26

**Authors:** Preeti Pancholi, Karen C. Carroll, Blake W. Buchan, Raymond C. Chan, Neelam Dhiman, Bradley Ford, Paul A. Granato, Amanda T. Harrington, Diana R. Hernandez, Romney M. Humphries, Matthew R. Jindra, Nathan A. Ledeboer, Shelley A. Miller, A. Brian Mochon, Margie A. Morgan, Robin Patel, Paul C. Schreckenberger, Paul D. Stamper, Patricia J. Simner, Nancy E. Tucci, Cynthia Zimmerman, Donna M. Wolk

**Affiliations:** a The Ohio State University Wexner Medical Center, Columbus, Ohio, USA; b The Johns Hopkins University School of Medicine, Baltimore, Maryland, USA; c Medical College of Wisconsin, Milwaukee, Wisconsin, USA; d Cedars-Sinai Medical Center, Los Angeles, California, USA; e Med Fusion, Lewisville, Texas, USA; f University of Iowa Hospitals and Clinics, Iowa City, Iowa, USA; g Laboratory Alliance of Central New York, Liverpool, New York, USA; h Loyola University Medical Center, Maywood, Illinois, USA; i Geisinger, Danville, Pennsylvania, USA; j UCLA, Los Angeles, California, USA; k Banner Gateway Medical Center, Gilbert, Arizona, USA; l Mayo Clinic, Rochester, Minnesota, USA; m MRIGlobal, Gaithersburg, Maryland, USA; Virginia Commonwealth University Medical Center

**Keywords:** rapid, FISH, identification, morphokinetic cellular analysis, phenotypic, antimicrobial susceptibility testing, MIC, blood culture, bacteremia, candidemia

## Abstract

We describe results from a multicenter study evaluating the Accelerate Pheno system, a first of its kind diagnostic system that rapidly identifies common bloodstream pathogens from positive blood cultures within 90 min and determines bacterial phenotypic antimicrobial susceptibility testing (AST) results within ∼7 h. A combination of fresh clinical and seeded blood cultures were tested, and results from the Accelerate Pheno system were compared to Vitek 2 results for identification (ID) and broth microdilution or disk diffusion for AST. The Accelerate Pheno system accurately identified 14 common bacterial pathogens and two Candida spp. with sensitivities ranging from 94.6 to 100%. Of fresh positive blood cultures, 89% received a monomicrobial call with a positive predictive value of 97.3%. Six common Gram-positive cocci were evaluated for ID. Five were tested against eight antibiotics, two resistance phenotypes (methicillin-resistant Staphylococcus aureus and Staphylococcus spp. [MRSA/MRS]), and inducible clindamycin resistance (MLSb). From the 4,142 AST results, the overall essential agreement (EA) and categorical agreement (CA) were 97.6% and 97.9%, respectively. Overall very major error (VME), major error (ME), and minor error (mE) rates were 1.0%, 0.7%, and 1.3%, respectively. Eight species of Gram-negative rods were evaluated against 15 antibiotics. From the 6,331 AST results, overall EA and CA were 95.4% and 94.3%, respectively. Overall VME, ME, and mE rates were 0.5%, 0.9%, and 4.8%, respectively. The Accelerate Pheno system has the unique ability to identify and provide phenotypic MIC and categorical AST results in a few hours directly from positive blood culture bottles and support accurate antimicrobial adjustment.

## INTRODUCTION

Bacteremia and candidemia associated with sepsis are major causes of morbidity and mortality worldwide. The conditions affect as many as 650 patients per 100,000 population, and the incidence has been increasing (1). Delayed administration of active antimicrobial agents to patients in septic shock is associated with a decrease in survival for every hour therapy is delayed (2). Early administration of active antimicrobials is therefore critical for improving outcomes and reducing mortality in patients with sepsis (3). Accurate and timely identification (ID) and antimicrobial susceptibility testing (AST) of the microorganism(s) causing sepsis are crucial to helping physicians select the most efficacious targeted therapy (4, 5).

Traditional ID and AST results for the microorganisms causing bloodstream infections can take 48 h or longer to obtain (6). Immediately after blood is collected for culture, empirical broad-spectrum antimicrobial therapy is initiated in patients suspected to have sepsis, and therapy is continued until the etiological agent is identified and AST results are available to tailor therapy (4). Studies show that many patients with community-acquired bacteremia, health care-associated bacteremia, and/or candidemia receive incorrect, inadequate, or excessively broad therapy during the empirical treatment period (4, 7). Incorrect continuous treatment with broad-spectrum antimicrobials can lead to drug toxicity, antimicrobial drug resistance, increased length of stay (LOS), including longer intensive care unit (ICU) stays, and additional costs for patients and the health care system (8, 9, 10). Inadequate empirical therapy is also associated with increased mortality (10). Furthermore, delays in microbial ID and AST may result in a delay in de-escalation of therapy from broad-spectrum to targeted antimicrobials.

Molecular diagnostic assays are now available for direct testing of positive blood cultures (BCs), providing timelier ID results. These tests detect multiple ID targets, characterizing >80% of positive blood cultures and providing accurate pathogen ID. Some systems additionally detect acquired resistance genes, such as *mecA*, *vanA* or *vanB*, *CTX-M*, and carbapenemase genes (11, 12). Known limitations of these molecular diagnostic tests include lack of sensitivity in detecting all organisms present in polymicrobial cultures and the limited susceptibility information (6, 13), as none of these produce a phenotypic MIC susceptibility result. Additionally, molecular assays are “add-on” tests, performed in addition to the required conventional phenotypic testing, and therefore increase the complexity of the laboratory workflow and the cost of patient care.

The Accelerate Pheno system for positive blood cultures changes this paradigm by combining ID and rapid phenotypic AST into one instrument. The system can provide ID within 90 min and AST results in approximately 7 h from a positive blood culture bottle, allowing health care personnel to evaluate phenotypic MIC susceptibility data to aid in the antibiotic escalation/de-escalation stewardship decisions. The Accelerate Pheno system uses an automated sample preparation and bacterial immobilization method to enable microscopy-based, single-cell analysis for ID and AST. Bacterial and candidal cell-by-cell ID is performed using fluorescence *in situ* hybridization (FISH). The MIC determination and susceptibility interpretation reports are generated using morphokinetic cellular analysis (MCA) by dark-field microscopy observation of individual, live, growing, immobilized bacterial cells in near real time (approximately every 10 min) in the presence (test) or absence (control) of a single concentration of antimicrobial agents. In this multicenter study, we compared results from the Accelerate Pheno system to those from a previously FDA-cleared semiautomated ID test system and triplicate broth microdilution (BMD) or disk diffusion for AST. A portion of the data generated in this study was used to support regulatory submissions for classification as an *in vitro* diagnostic (IVD) device.

## MATERIALS AND METHODS

### Study sites.

Thirteen geographically diverse U.S. clinical sites (Lewisville, TX; Iowa City, IA; Los Angeles, CA [2 sites]; Liverpool, NY; Rochester, MN; Milwaukee, WI; Columbus, OH; Gilbert, AZ; Maywood, IL; Danville, PA; Baltimore, MD; Tucson, AZ) enrolled and tested positive blood cultures (BCs) with the Accelerate Pheno system using the Accelerate PhenoTest BC kit. A reference laboratory (MRIGlobal, Palm Bay, FL) tested isolates sent from the clinical sites using reference/comparator methods.

### Overall design.

This study had two experimental arms and three phases. The sample pool included 50% fresh, patient deidentified, residual positive BC samples (prospective arm [*n* = 1,244]), and 50% isolates seeded into blood culture bottles injected with human blood (seeded arm [*n* = 1,256]). Institutional Review Board (IRB) approval and a waiver of informed consent were obtained at each site. Study phases and bottle types are described in the methods section in the supplemental material.

Only one prospective sample per patient was enrolled, and a minimum of 8 ml of each positive BC broth was required. Following enrollment, positive BC bottles were enrolled within 8 h after the positive result and assigned a unique study number. Gram staining was performed, and aliquots of the positive blood sample were submitted for routine standard of care (SoC) ID and AST testing at the local site, according to each laboratory's standard operating procedures. Fresh samples were deidentified prior to testing on the Accelerate Pheno system. Preparation of two, 1-ml positive BC aliquots for frozen stocks (−80°C) and plating of samples occurred within 8 h of positivity. Isolates from overnight plated samples were placed in transport medium (ESwab liquid Amies collection and transport system [Copan Diagnostics Inc. Murrieta, CA]) and shipped daily to the reference laboratory where the organisms were subcultured for ID and AST comparator testing. Quality control testing was performed by the reference laboratory on each day of testing. External controls with results that were outside the specified levels were repeated. If the repeated control was outside the specified levels, results were not reported for that organism and/or antimicrobial agent for that day.

Per IRB protocol, a designated person at each site recorded SoC ID and AST results for each study number. Accelerate Pheno system technical and assay failures were also recorded to determine system reliability.

**FIG 1:**
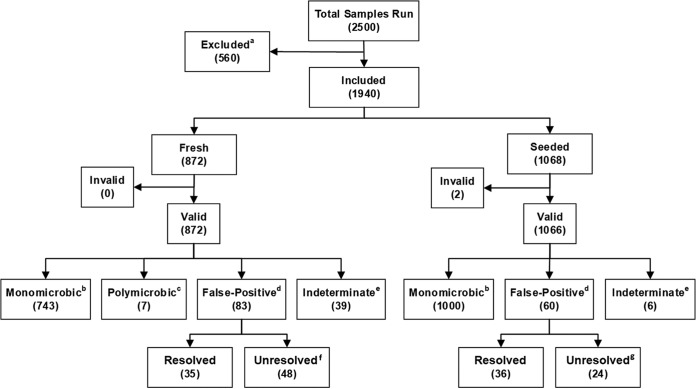
Flowchart of sample disposition after reevaluation of data with the 2017 software update. Footnotes a to g in the flowchart include additional information about the categories. For footnote a, the 560 excluded samples include the following reasons for exclusion: deviation from the protocol (*n* = 216), experiments halted (*n* = 26), experiments never run (*n* = 15), bottle received >8 h after positive result (*n* = 31), Gram staining shows no organism (*n* = 24), isolate not received at the reference laboratory (*n* = 18), isolate received more than 4 days after medium preparation (*n* = 3), ID reference growth failure (*n* = 29), nonpure isolate (*n* = 169), ID reference purity plate failure (*n* = 6), invalid ID reference result (*n* = 1), and Accelerate Pheno system run state not “complete” (*n* = 22). For footnote b with monomicrobic, a single on-panel organism was reported. For footnote c with polymicrobic, this category includes polymicrobial samples where the Accelerate PhenoTest BC kit ID results exactly match the reference results. For footnote d with false-positive, the false-positive category includes monomicrobial or polymicrobial runs containing any false-positive result(s). For footnote e with indeterminate, all indeterminate samples had only indeterminate/negative results. For footnote f with unresolved for fresh samples, of the 48 fresh unresolved false-positive results, 43 showed genus-level agreement, while the remaining five were one S. aureus called CoNS, one S. aureus plus Pantoea species mix called Klebsiella spp., one Pseudomonas putida called Citrobacter spp., one Lactococcus raffinolactis called Streptococcus spp., and one Streptococcus dysgalactiae subsp. equisimilis called E. faecium by the Accelerate Pheno system. For footnote g with unresolved for seeded samples, of the 24 seeded samples containing unresolved false-positive results, 15 showed genus-level agreement, one sample gave two false-positive results, while the remaining nine were one Pantoea sample called Enterobacter spp., one C. koseri sample called Citrobacter spp. plus Proteus spp., two C. koseri samples called Citrobacter spp. plus Klebsiella spp., one C. freundii sample called Citrobacter spp. plus Enterobacter spp., one C. koseri sample called Citrobacter spp. plus E. faecium plus Klebsiella spp., one E. cloacae complex sample called Enterobacter spp. plus P. aeruginosa, one E. faecalis sample called E. faecalis plus E. faecium, and one Streptococcus pyogenes sample called Streptococcus spp. plus E. faecium by the Accelerate Pheno system.

For seeded samples, more than one isolate per patient could be enrolled if the organism identification was different. Seeded organisms were derived from archived bacterial and yeast isolates that were cultured from positive BCs, and other clinical samples. Seeded cultures were prepared as described in the supplemental methods section. Once flagged positive by the automated blood culture instruments, the seeded positive cultures underwent the same testing as prospective samples (except for deidentification for isolates not derived from recent patient samples). Contaminated blood culture samples were excluded (Fig. 1).

### Accelerate PhenoTest BC kit testing and stock preparation.

Accelerate PhenoTest BC kit testing was performed using the Accelerate Pheno system according to the manufacturer's instructions (14). Briefly, the kit was removed from refrigerated storage, and the cassette, reagent cartridge, and sample vial were removed from packaging. Eight milliliters of positive BC broth was removed from the blood culture bottle, and 5 ml was loaded into the sample vial, 155 μl of which was used in the assay (the sample vial was updated for the FDA-cleared IVD device to require only 500 μl to be loaded). Before initiation of a run, the sample vial was placed in the reagent cartridge, which was placed in the Accelerate Pheno system, along with a test cassette. The instrument automatically performed sample cleanup, organism immobilization, FISH ID, and MCA-based AST, with ID results reported within 90 min and AST results reported within ∼7 h.

Bacterial ID and AST targets are shown in Tables S1 and S2 in the supplemental material with reportable ranges. The MICs are interpreted by the Accelerate Pheno system software, using FDA breakpoints (or Clinical and Laboratory Standards Institute [CLSI] in research use only [RUO] mode, where these differed). Expert rules in software mitigate false resistance or false susceptibility results. Yeast ID targets are Candida albicans and Candida glabrata. Detection of off-panel organisms was not claimed in regulatory submissions; however, they were included in the specificity analysis for identification of organisms. The system provides a monomicrobial call, which indicates that only one pathogen was detected in the sample.

### Reference laboratory comparator testing.

Isolates were subcultured by the reference laboratory within 4 days of inoculation onto transport medium at the clinical site. Only viable, pure isolates obtained from undamaged, properly labeled transport medium vials, under the appropriate transport and storage conditions underwent Gram staining and reference testing. Frozen isolate stocks (−80°C) were prepared from subcultured plates in cryopreservative vials containing Trypticase soy broth (TSB) and glycerol (MicroVial; Fisher Scientific, Hampton, NH) for discrepancy testing. The SoC ID results were used as the reference for Streptococcus species isolates that did not grow at the reference laboratory. Gram-positive rods, Gram-negative cocci, and anaerobes were excluded from reference testing. Isolates from polymicrobial samples were tested individually. The reference comparator for ID testing was the Vitek 2 instrument (bioMérieux; software version v07.01), performed per the manufacturer's instructions using the Vitek 2 GN ID card (catalog no. 21341), Vitek 2 GP ID card (catalog no. 21342), and Vitek 2 YST ID card (catalog no. 21343). Species-level identification via whole-genome sequencing (WGS) was performed on all Streptococcus spp. and Acinetobacter baumannii complex isolates to confirm ID results; WGS was performed using Illumina's MiSeq platform with a 2 × 151 paired-end protocol, using 300-cycle MiSeq reagent kits v2 and standard size flow cells. Results were analyzed using a proprietary algorithm (Accelerate Diagnostics, internal data).

The reference standard for AST comparator testing was Clinical and Laboratory Standards Institute reference frozen BMD and the reference standard for cefoxitin testing of staphylococci was disk diffusion. In both cases, triplicate BMD or disk testing was performed for each isolate (see supplemental methods section).

### Discrepancy testing.

False-negative ID results were defined as negative FISH ID probe results by the Accelerate Pheno system, and a positive, on-panel ID by the reference methods. False-negative results were retested in triplicate using frozen blood culture samples and the Accelerate Pheno system at Accelerate Diagnostics, Inc. If the retested samples still indicated a negative result, WGS as described above was performed to confirm the Vitek 2 ID result.

For AST, frozen isolates were created at the clinical sites as needed for discrepancy testing (see supplemental methods section). The isolate from the original blood culture bottle and the isolate submitted to the reference laboratory were respiked into separate bottles of the original blood bottle type at Accelerate Diagnostics, Inc. Bottles were incubated until they flagged positive; the resulting positive blood cultures were tested on the Accelerate Pheno system using the Accelerate PhenoTest BC kit, in triplicate, along with parallel triplicate BMD (see supplemental methods section). Samples for which more than one drug had a very major error (VME) for a single isolate were additionally tested using Vitek 2 (GP67 and GN82) and disk diffusion to confirm the results by a secondary method.

### Statistics.

For ID performance, R code version 3.3.2 was used to calculate sensitivity (positive percent agreement [PPA]) and specificity (negative percent agreement [NPA]) with 95% Wilson confidence intervals (15–17) for each FISH ID probe. For the purposes of accuracy reporting, both fresh and seeded samples were combined. A sufficient number of samples were tested for ID to establish the requisite lower confidence limit required by the FDA. The indeterminate (no result for a FISH ID probe) rate was calculated for each ID probe, and the overall invalid (no ID result for a sample) rate was calculated out of the total number of samples. The positive predictive value (PPV) for the monomicrobial call was also calculated before and after arbitration by Gram stain results. ID results as cleared by the FDA (February 2017, software version 1.2.1) and after a post-FDA clearance 2017 software update (service pack PSW000002 for version 1.2.1) were calculated. The software update modified interpretation of ID algorithm results. Only samples with valid results using both the test and reference methods were included in ID performance analysis.

For AST performance, BMD results were truncated to the same range as the investigational test results (i.e., Accelerate Pheno system). FDA breakpoints were used for all IVD organism/antimicrobial combinations. 2016 CLSI breakpoints were used for all RUO organism/antimicrobial combinations except for members of the family Enterobacteriaceae with colistin, which used 2016 EUCAST breakpoints. For antimicrobial agents that yielded an MIC result, essential agreement (EA) and categorical agreement (CA) were calculated. VME, major error (ME), and minor error (mE) rates were also calculated in certain cases. For resistance phenotype tests, only CA, ME, and VME rates were calculated (see supplemental methods section). Only samples with valid ID results by both methods, samples where the test ID matched the reference ID, and samples with valid AST results by both methods were included in the AST performance analysis. Samples with documented protocol deviations and quality control (QC) failures were excluded from analysis.

The study included a sufficient sample size to meet FDA requirements for both ID and AST. In some cases, more organisms were tested than required for determination of ID to reach statistical significance requirements for AST of some antimicrobials. Technical failure, ID invalid, and ID indeterminate results were excluded from performance analysis, but rates were calculated for reportability compared to the reference methods. Results with QC failures for individual probes and drugs were excluded.

## RESULTS

### Genus and species identification.

During the study, 2,500 positive BC bottles (seeded and fresh) were tested with the Accelerate Pheno system. In this study, the data for these 2,500 BCs were reanalyzed using the updated 2017 software. After analysis with the new software, 560 samples were excluded as listed in Fig. 1. Of the remaining 1,940 samples, 872 were fresh prospective samples, yielding 872 (100%) valid results, and 1,068 were seeded samples, with 1,066 (99.8%) valid results.

Within the sample set, 83/872 (9.5%) fresh prospective samples were classified as giving false-positive results (Fig. 1). However, 35 (4.0%) fresh samples were resolved by a demonstrated absence of organism by Gram staining (defined as mitigated by Gram staining in the Accelerate PhenoTest BC kit instructions for use [IFU] [14]). The remaining 48 (5.5%) fresh samples were unresolved. Of these 48 samples, 43 were found to generate correct results at the genus level, leaving only 5 truly false-positive samples that could not be resolved (0.6%) (Fig. 1). Note that this study uses a variation of the traditional term “false positive” and defines false-positive results broadly. For example, a Citrobacter braakii isolate that reacted with the Citrobacter species probe was classified as a false-positive result, because Citrobacter braakii is not a species that was originally included in the Accelerate PhenoTest BC kit claimed panel. Likewise, Staphylococcus cohnii and Staphylococcus simulans were classified as false-positive results when they reacted with the coagulase-negative staphylococcus (CoNS) probe based on FDA claims, despite being correctly identified as coagulase-negative staphylococci.

Similar information for the FDA software-cleared data is found in Fig. S1 in the supplemental material along with the Accelerate PhenoTest BC kit IFU (14); comparison of this publication with the IFU shows the impact and improvements derived from the 2017 software update. Briefly, for fresh samples in the FDA software-cleared data, 79 of 872 (9.1%) were invalid, and 27 (3.1%) included at least one indeterminate result. Reanalysis of these data with the postclearance 2017 software update successfully eliminated most of the indeterminate results from the FDA software-cleared data, as well as all 79 formerly invalid results (Fig. 1). However, additional indeterminate results were produced for the 79 newly valid samples, resulting in a final indeterminate rate of 39/872 (4.5%).

The outcomes of seeded samples, evaluated by the 2017 software update are also displayed in Fig. 1. There were 60/1,066 (5.6%) false-positive samples, 36 that were resolved by Gram stain results and 24 that were unresolved. Of the 24 unresolved results, 15 samples had correct results to the genus level, but with species not claimed in the FDA submission. The remaining nine unresolved samples are outlined in Fig. 1 footnotes. One sample had two false-positive results, with genus-level agreement for one of the two false-positive results.

In the supplemental material (Fig. S1) and in the IFU (14), accuracy testing of the Accelerate Pheno system versus reference standard was performed, and results at FDA clearance are listed as percentages followed by 95% confidence intervals (95% CI) in parentheses. At FDA clearance, the Accelerate PhenoTest BC kit identification performance had an overall sensitivity of 97.4% (95% CI, 96.5 to 98.0%) and specificity of 99.3% (95% CI, 99.2 to 99.4%).

After revisions to ID algorithm interpretations in the 2017 update to the Accelerate Pheno system software, invalid results were reduced with similar overall performance for microbial identification (Table 1). Observed overall sensitivity and specificity remained largely equivalent to the original performance of the FDA-cleared software (14), despite a slight increase to 97.5% (95% CI, 96.7 to 98.1%) and 99.5% (95% CI, 99.4 to 99.5%), respectively. When 2017 software results were substratified by Gram stain morphology, i.e., Gram-positive bacteria, Gram-negative bacteria, and yeasts (Table 1), the sensitivity was largely unchanged, 96.7% (95% CI, 95.4 to 97.7%), 98.5% (95% CI, 97.4 to 99.2%), and 97.9% (95% CI, 92.7 to 99.4%), respectively, and the specificity was slightly improved at 99.0% (95% CI, 98.8 to 99.2%), 99.8% (95% CI, 99.7 to 99.8%), and 99.6% (95% CI, 99.3 to 99.8%), respectively.

**TABLE 1:** Identification performance of Gram-positive bacteria, Gram-negative bacteria, and yeasts after reevaluation of DNA probe data with the 2017 Accelerate Pheno system software update^*a*^

Probe (category and species)	No. of samples with the following result^*b*^:				Sensitivity(%) (95% CI)^*c*^	Specificity (%) (95% CI)^*c*^
	TP	FN	TN	FP		
Gram-positive bacteria						
Staphylococcus aureus	242	5	1,643	19	98.0 (95.4–99.1)	98.9 (98.2–99.3)
CoNS^*d*^	264	15	1,589	28	94.6 (91.3–96.7)	98.3 (97.5–98.8)
Staphylococcus lugdunensis	77	2	1,857	1	97.5 (91.2–99.3)	100.0 (99.7–100)
Enterococcus faecium	109	4	1,809	9	96.5 (91.3–98.6)	99.5 (99.1–99.7)
Enterococcus faecalis	102	2	1,814	3	98.1 (93.3–99.5)	99.8 (99.5–99.9)
Streptococcus spp.	180	5	1,678	46	97.3 (93.8–98.8)	97.3 (96.5–98)
Total	974	33	10,390	106	96.7 (95.4–97.7)	99.0 (98.8–99.2)
Gram-negative bacteria						
Escherichia coli	148	2	1,771	2	98.7 (95.3–99.6)	99.9 (99.6–100)
Klebsiella spp.	126	3	1,790	6	97.7 (93.4–99.2)	99.7 (99.3–99.9)
Enterobacter spp.	108	2	1,822	4	98.2 (93.6–99.5)	99.8 (99.4–99.9)
Proteus spp.	88	1	1,838	6	98.9 (93.9–99.9)	99.7 (99.3–99.9)
Citrobacter spp.	95	1	1,768	8	99.0 (94.3–100)	99.6 (99.1–99.8)
Serratia marcescens	50	0	1,885	1	100.0 (92.9–100)	100.0 (99.7–100)
Pseudomonas aeruginosa	57	1	1,865	3	98.3 (90.9–99.9)	99.8 (99.5–100)
Acinetobacter baumannii	69	1	1,854	3	98.6 (92.3–99.9)	99.8 (99.5–100)
Total	741	11	14,593	33	98.5 (97.4–99.2)	99.8 (99.7–99.8)
Yeasts						
Candida albicans	44	1	1,827	7	97.8 (88.4–99.9)	99.6 (99.2–99.8)
Candida glabrata	49	1	1,818	8	98.0 (89.5–99.9)	99.6 (99.1–99.8)
Total	93	2	3,645	15	97.9 (92.7–99.4)	99.6 (99.3–99.8)
**Overall**	**1,808**	**46**	**28,628**	**154**	**97.5 (96.7–98.1)**	**99.5 (99.4–99.5)**

a The overall data for all organisms tested are shown in boldface type in the table.

b The test results are shown as follows: TP, true positive; FN, false negative; TN, true negative; FP, false positive. See the definitions for test results in the supplemental Materials section.

c Sensitivity and specificity are shown as percentages. The values in parentheses are the 95% confidence intervals.

d CoNS, coagulase-negative Staphylococcus spp.

When accuracy data were examined by ID probe, the 2017 Accelerate Pheno system software update produced a slight increase in sensitivity for Staphylococcus aureus, Enterococcus faecalis, Streptococcus spp., Escherichia coli, Klebsiella spp., Enterobacter spp., Proteus spp., and Citrobacter spp., while other organism groups remained the same or produced a slight decrease (Table 1). When assessing specificity, the 2017 software version produced results that either remained the same or produced a slight increase for all microbial groups except for Enterococcus spp. and Streptococcus spp. (Table 1).

Using the 2017Accelerate Pheno system software update for interpretation, 775/872 (89%) of fresh samples received a monomicrobial call, and of those, 754 (97.3%) were confirmed to be monomicrobial by reference testing (Table 2). Without resolving the result by the companion Gram stain of the blood culture, the PPV of the monomicrobial call result was 97.3% (95% CI, 95.9 to 98.2%); essentially 18 to 41 blood cultures in 1,000 could be a mixed culture but would have resulted in a monomicrobial call (Table 2). Importantly, when the blood culture broth Gram stain results were considered in addition to the monomicrobial result, the PPV rose to 99.4% (95% CI, 98.5 to 99.7%) (Table 2). In other words, 3 to 15 results in 1,000 would produce a false monomicrobial call and could represent a mixed infection. Specifically, there were 21 false-positive monomicrobial calls, of which 16 were resolved by Gram staining (Table 2). Of the remaining five, the presence of an additional organism not detected by the monomicrobial call included the following: one off-panel Streptococcus species had genus-level agreement with the positive Streptococcus call, two were CoNS, one was an off-panel viridans group Streptococcus species, and one was a Klebsiella pneumoniae in the presence of C. braakii.

**TABLE 2:** Monomicrobial call performance comparison with FDA-cleared Accelerate Pheno system software and postclearance 2017 software update

Parameter^*a*^	Value for software	
	FDA-cleared	2017 software update
No. of total valid samples	793	872
Total no. of MONO by Accelerate PhenoTest BC kit	557	775
Total no. of MONO by Accelerate PhenoTest BC kit and confirmed by reference	545	754
No. of false MONOs	12	21
No. of false MONO, resolved by Gram staining	10	16
No. of false MONO, unresolved by Gram staining	2	5
Positive predictive value (95% CI)	97.8 (96.3–98.8)	97.3 (95.9–98.2)
Positive predictive value after resolving with Gram staining (95% CI)	99.6 (98.7–99.9)	99.4 (98.5–99.7)

a MONO, monomicrobial calls. The positive predictive value is the percentage correct.

For indeterminate results, Table 3 depicts the data analyzed with the FDA-cleared Accelerate Pheno system software and compared to the 2017 software update. The Accelerate Pheno system 2017 software update lowered the percent indeterminate calls in most cases except for Streptococcus spp., E. coli, Proteus spp., and Pseudomonas aeruginosa, for which all results were negligibly increased and C. albicans and C. glabrata whose indeterminate rates slightly increased by 2.0% and 2.3%, respectively. Notably, fewer false-positive results were observed after the 2017 software update for the Candida probes, particularly for the C. glabrata probe (Table 1 and Table S3). Indeterminate rates were lowered for all bacterial identification groups with improvements as high as 3.7% for CoNS (from 5.9% to 2.2%), and a substantial decrease in indeterminate calls for Klebsiella spp., Enterobacter spp., Staphylococcus lugdunensis, and S. aureus (Table 3).

**TABLE 3:** Indeterminate identification results by DNA probe with FDA-cleared Accelerate Pheno system software and postclearance 2017 software update

Probe (category and species)	No. of indeterminate results (%) by software:	
	FDA-cleared^*a*^	2017 software update^*b*^
Gram-positive bacteria		
Staphylococcus aureus	35 (1.9)	29 (1.5)
CoNS^*c*^	110 (5.9)	42 (2.2)
Staphylococcus lugdunensis	22 (1.2)	1 (0.1)
Enterococcus faecium	6 (0.3)	4 (0.2)
Enterococcus faecalis	4 (0.2)	1 (0.1)
Streptococcus spp.	9 (0.5)	18 (0.9)
Gram-negative bacteria		
Escherichia coli	0 (0.0)	7 (0.4)
Klebsiella spp.	55 (3.0)	2 (0.1)
Enterobacter spp.	47 (2.5)	0 (0.0)
Proteus spp.	0 (0.0)	1 (0.1)
Citrobacter spp.	0 (0.0)	0 (0.0)
Serratia marcescens	0 (0.0)	0 (0.0)
Pseudomonas aeruginosa	0 (0.0)	9 (0.5)
Acinetobacter baumannii	0 (0.0)	0 (0.0)
Yeasts		
Candida albicans	6 (0.3)	44 (2.3)
Candida glabrata	0 (0.0)	44 (2.3)

a The results for FDA-cleared software are the total number of indeterminate results divided by the total number of valid tests per probe (1,850).

b The results for 2017 software update are the total number of indeterminate results divided by the total number of valid tests per probe (1,940).

c CoNS, coagulase-negative Staphylococcus spp.

When an alternate classification approach was used, one that considers indeterminate results by sample and not by probe result (Fig. 1 and Table S4), the overall indeterminate rate for the 2017 software update was 2.3% (45/1,938), ranging from 0.6% (6/1,066) in seeded samples to 4.5% (39/872) in fresh samples. The final overall invalid rate was 0.1% (2/1,940) ranging from 0% (0/872) in fresh samples to 0.2% (2/1,068) in seeded samples.

### Gram-positive AST results.

The cumulative AST data for the Gram-positive pathogens, including RUO combinations, are displayed in Table 4 by organism group and antimicrobial agent. In total, 4,142 AST results from the different organism/antimicrobial combinations were obtained in an average of 6.47 h. The overall EA and CA were 97.6% (range, 89.7 to 100%; 95% CI, 97.1 to 98.1%) and 97.9% (range, 87.1 to 100%; 95% CI, 97.5 to 98.3%), respectively. Overall VME, ME, and mE rates were 1.0% (95% CI, 0.5 to 1.9%), 0.7% (95% CI, 0.4 to 1.0%) and 1.3% (95% CI, 1.0 to 1.7%), respectively.

**TABLE 4:** Gram-positive AST results by organism/antimicrobial combination^*a*^

Class or parameter	Antimicrobial agent or organism	Organism	EA (%)	CA (%)	No. of samples:			No. of samples with the following AST result^*b*^:			No. of errors:		
					Test	Fresh	Seed	S	I	R	VME	ME	mE
Penicillins	Ampicillin	EFM	100	99	96	44	52	4	0	92	0	1	0
		EFS	100	100	142	134	8	142	0	0	0	0	0
Cephems	Ceftaroline	SAU	93.3	99.7	344	297	47	343	1	0	0	0	1
Lipopeptides	Daptomycin	SAU	98.5	99.5	197	148	49	196	0	1	1	0	0
		SLU^*c*^	96.6	100	29	1	28	29	0	0	0	0	0
		CoNS	100	100	135	135	0	135	0	0	0	0	0
		EFM	93	98.6	71	19	52	71	0	0	0	1	0
		EFS	100	100	40	32	8	40	0	0	0	0	0
Tetracyclines	Doxycycline^*c*^	SAU	96.9	97.7	383	336	47	378	5	0	0	4	5
		SLU	100	100	29	1	28	29	0	0	0	0	0
		CoNS	99.3	96.3	134	134	0	128	6	0	0	1	4
		EFM	99.2	87.1	124	69	55	83	5	36	0	0	16
Macrolides	Erythromycin	SAU	98.2	96.8	338	292	46	132	1	205	0	1	10
		SLU^*c*^	100	100	28	1	27	25	0	3	0	0	0
		CoNS^*c*^	97	95.5	134	134	0	40	1	93	1	0	5
Oxazolidinones	Linezolid	SAU	99.5	100	194	147	47	194	0	0	0	0	0
		SLU^*c*^	100	100	29	1	28	29	0	0	0	0	0
		CoNS^*c*^	100	100	135	135	0	135	0	0	0	0	0
		EFM	98.6	97.1	69	17	52	67	1	1	0	0	2
		EFS	92.7	100	41	33	8	41	0	0	0	0	0
Sulfonamide	TMP-SMX^*c*^	SAU	98.2	98.2	386	338	48	384	0	2	0	7	0
		SLU	89.7	89.7	29	1	28	29	0	0	0	3	0
Glycopeptide	Vancomycin	SAU	98	99	198	148	50	196	2	0	0	0	2
		SLU	100	100	29	1	28	29	0	0	0	0	0
		CoNS	100	100	134	134	0	134	0	0	0	0	0
		EFM	90.1	90.1	71	19	52	16	0	55	0	0	7
		EFS	92.7	92.7	41	33	8	36	0	5	0	0	3
Resistant phenotype	MRSA/MRS (cefoxitin)	SAU	N/A	99.5	184	141	43	86	N/A	98	0	1	N/A
		SLU	N/A	100	28	1	27	28	N/A	0	0	0	N/A
		CoNS	N/A	96.8	186	115	71	38	N/A	148	5	1	N/A
	MLSb (erythromycin-clindamycin)	SLU	N/A	100	29	1	28	27	N/A	2	0	0	N/A
		CoNS	N/A	97.8	135	135	0	67	N/A	68	1	2	N/A
		All	97.6	97.9	4142	2132	2010	3311	22	809	8	22	55

a Abbreviations: TMP-SMX, trimethoprim-sulfamethoxazole; MRSA, methicillin-resistant S. aureus; MRS, methicillin-resistant Staphylococcus spp.; MLSb, inducible clindamycin resistance; CoNS, coagulase-negative Staphylococcus spp.; EFM, Enterococcus faecium; EFS, Enterococcus faecalis; SAU, Staphylococcus aureus; SLU, Staphylococcus lugdunensis; N/A, not available.

b Abbreviations: S, susceptible; I, intermediate; R, resistant.

c Research use only (RUO).

Vancomycin was evaluated for Staphylococcus spp. (*n* = 361) and Enterococcus spp. (*n* = 112). All staphylococci tested were vancomycin susceptible (MIC range, ≤ 0.5 to 2 μg/ml), except for two S. aureus isolates that were intermediate (MIC, 4 μg/ml). For these two intermediate isolates, the Accelerate Pheno system produced MICs of 1 μg/ml and 2 μg/ml, resulting in a susceptible result. Of the enterococci, 60 were vancomycin resistant. Vancomycin EA and CA ranged from 98 to 100% for staphylococci and from 90.1 to 92.7% for enterococci. There were no MEs or VMEs. There were two mEs with S. aureus (Accelerate Pheno system susceptible, BMD intermediate), seven with Enterococcus faecium, and three with Enterococcus faecalis (all enterococci Accelerate Pheno system intermediate, BMD resistant). Daptomycin was evaluated for Staphylococcus and Enterococcus spp. with EA and CA ranging from 93 to 100% compared to the reference BMD method. Of the 472 results, only one S. aureus tested was daptomycin nonsusceptible. There was one VME with S. aureus (Accelerate Pheno system MIC of 0.5 μg/ml and BMD MIC of ≥2 μg/ml) and one ME with E. faecium (Accelerate Pheno system MIC of ≥8 μg/ml and BMD MIC of 2 μg/ml) (Table 5). Linezolid was evaluated for Staphylococcus and Enterococcus spp., with EA and CA ranging from 99.5 to 100% for staphylococci and 92.7 to 100% for enterococci. Of the 468 results, all samples tested susceptible by BMD except for one linezolid-intermediate and one resistant E. faecium, which both gave correct results by the Accelerate Pheno system (Table 4). There were two mEs with E. faecium, but no VME or ME for any of the species tested. Doxycycline was evaluated for Staphylococcus spp. and E. faecium with all EA and CA above 96%, except for the E. faecium CA of 87.1%. E. faecalis was also tested with doxycycline, but performance was below FDA acceptance criteria, and therefore, this combination was not included in the final product (data not shown). There were 25 mEs (16 for E. faecium isolates, 5 for S. aureus isolates, and 4 for CoNS isolates) and 5 MEs (4 for S. aureus isolates and 1 for a CoNS isolate), but no VME (Table 4) for doxycycline. Erythromycin EA and CA ranged from 95.5 to 100% for all Staphylococcus spp. evaluated. There was one VME (CoNS) and one ME (S. aureus) encountered (Table 4). For ceftaroline, of the 344 S. aureus isolates tested, all tested susceptible by BMD, except for one intermediate isolate that tested susceptible by the Accelerate Pheno system (Accelerate Pheno system MIC of 1 μg/ml and BMD MIC of 2 μg/ml). Overall, ceftaroline showed 93.3% EA and 99.7% CA. There were no MEs or VMEs (Table 4). For ampicillin, the 238 Enterococcus isolates evaluated showed excellent agreement with the reference BMD method with all EA and CA at 99% or above. There was only one ME with the ampicillin-E. faecium combination (Table 4). For trimethoprim-sulfamethoxazole (TMP-SMX), of the 415 staphylococcal samples tested, all were susceptible except for two resistant S. aureus samples. EA and CA for TMP-SMX for S. aureus were both 98.2%, while EA and CA for TMP-SMX for S. lugdunensis were both 89.7%. There were 10 MEs (7 for S. aureus and 3 for S. lugdunensis) encountered in TMP-SMX testing.

**TABLE 5:** Very major and major error summary by Gram-positive organism/antimicrobial combination

Error type and antimicrobial	Organism	No. of errors	MIC^*a*^		AST result^*b*^		Breakpoint^*a*^		Reportable range^*a*^	
			AXDX	REF	AXDX	REF	S	R	Low	High
Very major errors										
Daptomycin	Staphylococcus aureus	1	0.5	≥2	S	NS	1	N/A	0.25	2
Erythromycin	Staphylococcus warneri^*c*^	1	0.25	≥16	S	R	0.5	8	0.125	16
Cefoxitin (MRSA/MRS)	Staphylococcus epidermidis	4	N/A	N/A	NEG	POS	4	8	4	8
	Staphylococcus haemolyticus	1	N/A	N/A	NEG	POS	4	8	4	8
Erythromycin-clindamycin (MLSb)	Staphylococcus warneri	1	N/A	N/A	NEG	POS	2	4	2	4
Major errors										
Ampicillin	Enterococcus faecium	1	16	8	R	S	8	16	2	32
Daptomycin	Enterococcus faecium	1	≥8	2	NS	S	4	N/A	1	8
Doxycycline^*c*^	Staphylococcus hominis subsp. *hominis*	1	16	4	R	S	4	16	1	32
	Staphylococcus aureus	4	≥32	2	R	S	4	16	1	32
Erythromycin	Staphylococcus aureus	1	≥16	0.25	R	S	0.5	8	0.125	16
Cefoxitin (MRSA/MRS)	Staphylococcus epidermidis	1	N/A	N/A	POS	NEG	4	8	4	8
	Staphylococcus aureus	1	N/A	N/A	POS	NEG	4	8	4	8
Erythromycin-clindamycin (MLSb)	Staphylococcus epidermidis	1	N/A	N/A	POS	NEG	2	4	2	4
	Staphylococcus warneri	1	N/A	N/A	POS	NEG	2	4	2	4
TMP-SMX^*c*^^,^^*d*^	Staphylococcus aureus	1	≥8	2	R	S	2	4	0.5	8
		2	≥8	≤0.5	R	S	2	4	0.5	8
		2	4	1	R	S	2	4	0.5	8
		2	4	≤0.5	R	S	2	4	0.5	8
	Staphylococcus lugdunensis	3	4	≤0.5	R	S	2	4	0.5	8

a Antimicrobial values (MIC, breakpoint for susceptibility [S] and resistance [R], and reportable range) are given in micrograms per milliliter. AXDX, Accelerate Pheno system; REF, reference; N/A, not available.

b AST results are shown as follows: S, susceptible; NS, not susceptible; R, resistant; NEG, negative; POS, positive.

c RUO.

d TMP-SMX, trimethoprim-sulfamethoxazole.

### Resistance phenotype testing for MRSA/MRS and MLSb.

Both resistant phenotype tests (methicillin-resistant Staphylococcus aureus and Staphylococcus spp. [MRSA/MRS] [cefoxitin] and inducible clindamycin resistance [MLSb] [erythromycin-clindamycin]) showed >96% agreement with all organisms tested. For S. aureus (MRSA or methicillin-susceptible S. aureus [MSSA]) with cefoxitin, there were 184 total results (86 susceptible and 98 resistant), with 99.5% CA, one ME and no VME. For CoNS (excluding S. lugdunensis) and cefoxitin, there were 186 total results (38 susceptible and 148 resistant), with 96.8% CA with one ME and five VMEs (4 for Staphylococcus epidermidis, 1 for Staphylococcus haemolyticus). Discrepancy testing resolved one of the five VMEs. For S. lugdunensis and cefoxitin, there were 28 total results with 100% CA (all were susceptible; Table 4). Results for cefoxitin met all AST acceptance criteria for all organisms tested.

For the 135 CoNS isolates tested (67 susceptible and 68 resistant) for inducible clindamycin resistance (MLSb), there was 97.8% CA with two MEs and one VME. For the 29 S. lugdunensis isolates tested for MLSb, there was 100% CA (Table 4). Results for MLSb with CoNS and S. lugdunensis met all AST acceptance criteria. The ability of the Accelerate PhenoTest BC kit to test S. aureus with MLSb was not claimed because of high VME (5.2%) and ME (4.8%) rates which were outside FDA acceptance criteria.

A summary of the VMEs and MEs along with the breakpoints and reportable ranges for the antimicrobial agents between the reference and the Accelerate Pheno system results are presented in Table 5. Overall, there were eight VMEs for the Gram-positive MIC and phenotypic susceptibilities (one each with S. aureus and daptomycin, CoNS and erythromycin, and CoNS and MLSb and five for CoNS and cefoxitin). There were 22 MEs among the Gram-positive organisms, most of which were with S. aureus and TMP-SMX (*n* = 7), S. aureus and doxycycline (*n* = 4), and S. lugdunensis and TMP-SMX (*n* = 3). There were four MEs for the resistance phenotype tests (one each for CoNS and S. aureus with cefoxitin and two for CoNS with MLSb). Overall, the FDA criteria for acceptability were met or exceeded.

### Gram-negative AST results.

The cumulative AST data for the Gram-negative pathogens, including RUO combinations, are displayed in Table 6 by organism group and specific antimicrobial agent. In total, 6,331 AST results from the different organism/drug combinations were evaluated. The overall EA and CA were 95.4% (range, 80.9 to 100%; 95% CI, 94.9 to 95.9%) and 94.3% (range, 80.9 to 100%; 95% CI, 93.8 to 94.9%), respectively.

**TABLE 6:** Gram-negative AST results by organism/antimicrobial combination

Class	Antimicrobial	Organism^*a*^	EA (%)	CA (%)	No. of samples:			No. of samples with the following AST result:			No. of errors		
					Test	Fresh	Seed	S	I	R	VME	ME	mE
Aminoglycoside	Amikacin	Enteric	95.6	95.0	343	167	176	321	17	5	0	0	17
		PAE	97.6	100	42	12	30	31	0	11	0	0	0
		ABA	80.9	80.9	47	3	44	12	2	33	0	0	9
	Gentamicin	Enteric	99.7	99.7	343	177	166	293	3	47	0	0	1
		PAE	95.2	88.1	42	12	30	30	4	8	0	1	4
	Tobramycin	Enteric	96.0	96.0	347	179	168	284	11	52	0	0	14
		PAE	100	97.6	42	12	30	30	1	11	0	0	1
Carbapenems	Ertapenem	Enteric	98.9	98.6	351	181	170	316	6	29	0	2^*c*^	3
	Meropenem	Enteric	97.8	98.1	364	180	184	329	0	35	0	4^*d*^	3
		PAE	90.2	90.2	51	12	39	26	0	25	0	1	4
		ABA^*b*^	96.8	96.8	156	3	153	60	3	93	0	2	3
Cephalosporin	Cefazolin	Enteric^*b*^	95.3	85.8	274	144	130	131	27	116	0	0	39
	Cefepime	Enteric	97.7	96.9	349	180	169	280	6	63	1	0	10
		PAE	92.9	92.9	42	12	30	23	0	19	0	3	0
		ABA^*b*^	87.1	83.9	155	3	152	47	22	86	0	0	25
	Ceftazidime	Enteric	93.9	93.9	377	175	202	266	3	108	0	0	23
		PAE	90.6	88.7	53	12	41	25	0	28	0	6	0
	Ceftriaxone	Enteric	95.1^*e*^	96.6	324	166	158	215	2	107	0	0	11
Fluoroquinolone	Ciprofloxacin	Enteric	98.9	98.3	352	181	171	262	3	87	0	0	6
		PAE	92.9	97.6	42	12	30	28	0	14	0	0	1
		ABA^*b*^	96.8	98.1	155	3	152	51	1	103	0	0	3
Monobactam	Aztreonam	Enteric	96.6	97.7	348	179	169	257	3	88	1	1	6
Penicillin inhibitor	Ampicillin-Sulbactam	Enteric	92.2	84.2^*g*^	322	155	167	165	36	121	1	1	49
		ABA^*b*^	93.6	84.1	157	3	154	65	19	73	0	2	23
	Piperacillin-Tazobactam	Enteric	92.5	93.0	402	174	228	304	18	80	1	3^*f*^	24
		PAE	90.0	82.9	70	12	58	35	4	31	0	1	11
		ABA	97.9	97.9	47	3	44	5	0	42	0	1	0
Polymyxin	Colistin^*b*^	Enteric	93.3	97.9	329	152	177	314	0	15	3	4	0
		PAE	100	100	42	12	30	42	0	0	0	0	0
		ABA	90.4	91.9	136	3	133	132	0	4	1	10	0
Tetracycline	Minocycline^*b*^	ABA	97.4	92.1	227	3	224	198	12	17	0	1	17
All	All	All	95.4	94.3	6,331	2,522	3,809	4,577	203	1,551	8	43	307

a Abbreviations: PAE, Pseudomonas aeruginosa; ABA, Acinetobacter baumannii complex.

b RUO.

c Enterobacter species major error rate, 2/26 (7.7%).

d Enterobacter species major error rate, 3/39 (7.7%).

e S. marcescens essential agreement, 33/40 (82.5%).

f Klebsiella species major error rate, 2/45 (4.4%).

g Low categorical agreement for ampicillin-sulbactam with enteric bacteria was due to minor errors.

There were a total of 1,551 resistant organisms, among which there were eight false-susceptible results for an overall VME rate of 0.5% (95% CI, 0.3 to 1.0%). The overall ME rate was 0.9% (95% CI, 0.7 to 1.3%), and the mE rate was 4.8% (95% CI, 4.4 to 5.4%). Table 7 lists the specific organism/antimicrobial combinations for the VME and ME which are discussed in more detail below.

**TABLE 7:** Very major and major error summary by Gram-negative organism/antimicrobial combination

Error type and antimicrobial	Organism	No. of errors	MIC^*a*^		AST result^*b*^		Breakpoint^*a*^		Reportable range^*a*^	
			AXDX	REF	AXDX	REF	S	R	Low	High
Very major errors										
Aztreonam	Escherichia coli	1	2	16	S	R	4	16	1	32
Colistin^*c*^	Acinetobacter baumannii	1	1	4	S	R	2	4	0.5	8
	Escherichia coli	1	1	4	S	R	2	4	0.5	8
	Enterobacter cloacae complex	2	≤0.5	≥8	S	R	2	4	0.5	8
Cefepime	Escherichia coli	1	≤1	16	S	R	2	16	1	32
Ampicillin-sulbactam	Proteus mirabilis	1	≤4	32	S	R	8	32	4	64
Piperacillin-tazobactam	Escherichia coli	1	8	128	S	R	16	128	4	256
Major errors										
Aztreonam	Enterobacter aerogenes	1	16	4	R	S	4	16	1	32
Ceftazidime	Pseudomonas aeruginosa	1	16	8	R	S	8	16^*d*^	2	32
		3	16	≤2	R	S	8	16^*d*^	2	32
		1	≥32	8	R	S	8	16	2	32
		1	≥32	≤2	R	S	8	16	2	32
Colistin^*c*^	Acinetobacter baumannii	3	≥8	≤0.5	R	S	2	4	0.5	8
		5	4	≤0.5	R	S	2	4	0.5	8
		2	4	1	R	S	2	4	0.5	8
	Escherichia coli	2	≥8	≤0.5	R	S	2	4	0.5	8
	Enterobacter aerogenes	2	≥8	≤0.5	R	S	2	4	0.5	8
Ertapenem	Enterobacter aerogenes	2	2	0.5	R	S	0.5	2	0.125	4
Cefepime	Pseudomonas aeruginosa	2	16	≤2	R	S	8	16^*d*^	2	32
		1	≥32	8	R	S	8	16	2	32
Gentamicin	Pseudomonas aeruginosa	1	16	2	R	S	4	16	1	32
Meropenem	Acinetobacter baumannii	1	8	2	R	S	2	8	0.5	16
		1	8	1	R	S	2	8	0.5	16
	Escherichia coli	1	4	≤0.25	R	S	1	4	0.25	8
	Enterobacter aerogenes	1	4	≤0.5	R	S	1	4	0.5	8
		2	≥8	≤0.5	R	S	1	4	0.5	8
	Pseudomonas aeruginosa	1	8	≤1	R	S	2	8	1	16
Minocycline^*c*^	Acinetobacter baumannii	1	16	4	R	S	4	16	1	32
Ampicillin-sulbactam	Acinetobacter baumannii	1	32	4	R	S	8	32	2	64
		1	32	8	R	S	8	32	2	64
	Klebsiella oxytoca	1	32	8	R	S	8	32	2	64
Piperacillin-tazobactam	Acinetobacter baumannii	1	≥256	16	R	S	16	128	4	256
	Enterobacter aerogenes	1	128	16	R	S	16	128	4	256
	Klebsiella pneumoniae	2	128	16	R	S	16	128	4	256
	Pseudomonas aeruginosa	1	128	≤8	R	S	16	128	8	256

a Antimicrobial values (MIC, breakpoint for susceptibility [S] and resistance [R], and reportable range) are given in micrograms per milliliter. AXDX, Accelerate Pheno system; REF, reference.

b AST results are shown as follows: S, susceptible; R, resistant.

c RUO.

d These results become minor errors when CLSI breakpoints are applied.

### AST data for the Enterobacteriaceae.

Overall aminoglycoside EA and CA for the Enterobacteriaceae were ≥95%. There were no VMEs or MEs for this class of antibiotics. There was one mE for gentamicin and 14 and 17 mEs, for tobramycin and amikacin, respectively. Among the tobramycin mEs, seven (50%) were with E. coli isolates, and the Accelerate PhenoTest BC kit MICs were lower than the BMD MICs for six of the seven isolates. Among the 17 mEs for amikacin, 9 were with Klebsiella spp., 7 were with Enterobacter spp., and 1 was with Serratia marcescens. Overall EA and CA for the carbapenems ranged from 97.8 to 98.9%. Carbapenem resistance among the fresh clinical Enterobacteriaceae in the study was very low (0.6%). Among the 181 valid fresh clinical isolates, only one K. pneumoniae isolate was resistant to both ertapenem and meropenem. During the seeded phases of the study, 35 meropenem-resistant isolates were added, 27 of which were also resistant to ertapenem (Table 6). Two additional seeded isolates were ertapenem resistant but meropenem susceptible. No VMEs were observed for the carbapenems. That said, even after supplementation with challenge strains, the ability of the Accelerate Pheno system to detect ertapenem resistance among Citrobacter spp., Proteus spp., and S. marcescens and to detect meropenem resistance among these organisms and E. coli is unknown on the basis of available data. There were two ertapenem MEs for Enterobacter aerogenes isolates (2/26 [7.7%]), three meropenem MEs for Enterobacter spp. (3/39 [7.7%]), and one meropenem ME for an E. coli isolate. The ertapenem MIC values for the two MEs were 2 doubling dilutions higher than the reference MIC value; the differences for meropenem exceeded 2 doubling dilutions for all four MEs.

Variable results were observed among the four cephalosporin agents tested. Because cefazolin data were not submitted to FDA, there are currently no official claims for this agent on the Accelerate Pheno system. In this study, when cefazolin performance was analyzed using CLSI breakpoints, EA was 95.3% and no VMEs or MEs were observed. However, the CA for cefazolin when testing Enterobacteriaceae isolates was 85.8% due to 39 mEs (14.2%).

The overall EA and CA for ceftriaxone were 95.1% and 96.6%, respectively. However, for S. marcescens, EA was only 82.5% (33/40). Seven mEs were encountered, and therefore, MIC results for ceftriaxone with S. marcescens should be confirmed by another method.

The EA and CA for ceftazidime were both 93.9%. Twenty-three mEs were observed. In general, ceftazidime MIC values tended to be 1 doubling dilution higher than the reference BMD MIC mode (Table S5).

Testing of cefepime revealed high concordance (EA and CA, 97.7% and 96.9%, respectively). One VME was observed for an E. coli isolate tested during the fresh clinical phase. This isolate had a BMD MIC mode of 16 μg/ml and an Accelerate Pheno system MIC of ≤1 μg/ml. No MEs were observed, and 10 mEs distributed among several species were observed for this drug.

Ciprofloxacin is the sole fluoroquinolone on the panel, and the data for the Enterobacteriaceae showed very high EA (98.9%) and CA (98.3%), and only six mEs. In all cases, the Accelerate Pheno system MICs were higher than the modal BMD values.

For aztreonam, the Accelerate Pheno system EA was 96.6% and CA was 97.7%. One VME, one ME, and six mEs were observed. The VME occurred for an E. coli isolate with an MIC of 16 μg/ml by BMD that tested susceptible by the Accelerate Pheno system (MIC of 2 μg/ml). The ME occurred with one of the Enterobacter isolates with an MIC of 16 μg/ml that had an MIC of 4 μg/ml when tested by the reference method. Aztreonam MIC values tended to be 1 doubling dilution higher than the reference MIC value. Four of the six mEs occurred with E. coli, but there was no consistent trend compared to BMD.

Ampicillin-sulbactam had an EA of 92.2% and a CA of 84.2%, largely due to 49 mEs (26 with E. coli and 19 with Klebsiella spp.). There was one VME with an isolate of Proteus mirabilis (Accelerate Pheno system MIC of ≤4 μg/ml and BMD MIC of 32 μg/ml) and one ME for a Klebsiella oxytoca isolate (Accelerate Pheno system MIC of 32 μg/ml and BMD MIC of 8 μg/ml) when tested with this antibiotic. Ampicillin-sulbactam MIC values tended to be 1 doubling dilution higher by the Accelerate Pheno system than the reference MIC value. The performance for piperacillin-tazobactam demonstrated EA and CA of 92.5% and 93%, respectively. One VME (E. coli isolate with an Accelerate Pheno system MIC of 8 μg/ml and BMD MIC of 128 μg/ml) and three MEs were observed. The MEs were seen with two Klebsiella isolates and one Enterobacter isolate. The Accelerate Pheno system MIC was 128 μg/ml and the BMD MIC results were 16 μg/ml for all three isolates.

Colistin has an RUO designation due to a lack of an FDA indication for use with this group of organisms. Overall EA was 93.3% and CA was 97.9%. Few resistant isolates (*n* = 15) were tested; consequently, the VME rate (3/15 [20%]) was high.

### Pseudomonas aeruginosa AST.

Seventy P. aeruginosa isolates were tested (Table 6), most of which were seeded (*n* = 58). Performance for the aminoglycosides revealed EA of 97.6%, 100%, and 95.2% for amikacin, tobramycin, and gentamicin, respectively. CA was 100%, 97.6%, and 88.1% for amikacin, tobramycin, and gentamicin, respectively. There were no aminoglycoside VMEs, but there was one ME for gentamicin for an isolate with a BMD MIC of 2 μg/ml and an Accelerate Pheno system MIC of 16 μg/ml. A total of five mEs (4%) were noted, four for gentamicin and one for tobramycin. Meropenem EA and CA were both 90.2%. No VMEs were noted among the 25 resistant P. aeruginosa isolates tested (3 fresh, 22 seeded). One ME and 4 mEs were observed. Ceftazidime CA was 88.7% and EA was 90.6%, and the EA and CA results for cefepime were both 92.9%. No VMEs were observed for either drug. Six MEs were observed for ceftazidime. Note that the CLSI breakpoints include an intermediate category, whereas the FDA breakpoints do not, and of the six MEs, four were classified as mEs by CLSI standards. Three MEs were also seen when testing cefepime. Like ceftazidime, no intermediate category exists for this organism by FDA breakpoints, whereas there is an intermediate category by CLSI. As was the case for ceftazidime, two of the three MEs were mEs by CLSI breakpoints (Table 7). Eleven mEs (11/70 [15.7%]) resulted in a lower CA for P. aeruginosa and piperacillin-tazobactam (82.9%). The EA was 90%; there were no VMEs and only one ME. Data for the 42 P. aeruginosa isolates tested against colistin agreed 100% with the BMD results; however, there were no resistant isolates tested for an accurate assessment of VME.

### AST data for Acinetobacter baumannii.

Only three fresh prospective A. baumannii samples were encountered in the trial; therefore, the numbers were supplemented with 228 seeded samples. The EA and CA for amikacin were both 80.9%, related to nine mEs (Table 6). Cefepime EA was 87.1% and CA was 83.9%. The EA for ampicillin-sulbactam was 93.6% and CA was 84.1% related to 23 mEs. Of the 23 mEs, 15 were false-resistant results, and one was a false-susceptible result. For the remaining agents tested, EA and CA for meropenem, ciprofloxacin, and piperacillin-tazobactam ranged from 96.8 to 98.1%, while the EA and CA for colistin and minocycline ranged from 90.4 to 97.4%. Only one VME was seen for A. baumannii, and that was with colistin. However, 10 of the 16 MEs occurred with colistin. There was one ME out of five piperacillin-tazobactam-susceptible A. baumannii isolates, so a resistant result requires confirmation (Table S5). A total of 227 A. baumannii isolates were tested against minocycline. EA and CA were above 92%, and there were no VMEs.

Due to insufficient numbers of resistant isolates observed during the prospective study and despite attempts to supplement the data with challenge isolates, confirmation testing is suggested for several organism/antimicrobial combinations as summarized in Table S5.

### AST exclusions.

Of the 46/1,170 (2.6%) samples that produced AST results when the test ID did not match the reference ID that were excluded from AST performance calculations, 16 were resolved by Gram staining and 23 had genus-level agreement. This left seven samples (0.4%), five of which had a suspected incorrect reference result. Of the remaining two samples, one was an S. aureus isolate called CoNS by the Accelerate Pheno system. Ceftaroline was not tested, but all other tested antimicrobial agents agreed with the reference results. The other sample was S. aureus with Pantoea spp., which was called Klebsiella spp. by the Accelerate Pheno system. The Accelerate Pheno system tested the 14 Gram-negative antimicrobials for Enterobacteriaceae, which is appropriate for Pantoea spp., but BMD was not performed on the Pantoea isolate, so a comparison could not be made.

## DISCUSSION

Given the severity of bloodstream infections and the challenges of treatment due to increasing rates of antimicrobial resistance, rapid ID and faster determination of antimicrobial susceptibility of microbes are increasingly important to meet patients' clinical needs (18–22), particularly for high-risk patient groups (18, 19). Because traditional phenotypic methods often require several days for ID, molecular techniques (11, 12, 23–29) and matrix-assisted laser desorption/ionization time of flight mass spectrometry (MALDI-TOF MS) (30–34) are available to test positive blood culture broth, subsequently reducing microbial ID time with demonstrated accuracy to detect a variety of microbes (27–29, 35–43). The Accelerate Pheno system identifies pathogens in a time frame that is similar to those of automated molecular methods. On the basis of the high sensitivity of the ID, the Accelerate Pheno system can be performed in concert with Gram staining, as opposed to methods that require Gram staining prior to cartridge selection, thereby reducing the wait time before beginning the run. The simple workflow (∼2 min to load) makes testing during all three shifts possible in both large and small hospitals. Since only a single sample can be run on an instrument and it takes 7 h to complete, multiple instruments will be required if additional samples need to be tested.

The performance of the Accelerate Pheno system is on par with or exceeds other molecular systems for ID of bloodstream pathogens (24, 34, 35). ID by the Accelerate Pheno system was robust compared to the reference methods and was obtained within 90 min. Although in some cases, organisms within the same genus as the detecting probe were classified as “false positives,” this terminology applied to species that were not included in the specific probe claim, such as certain species of Streptococcus and CoNS (refer to the table in the supplemental methods section for the list of species on the panel). When the 2017 software update was used for analysis, accurate classification of positive and negative results occurred for 30,226 of 30,426 results (total agreement, 99.3%) in a sample set in which fresh samples accounted for 50% of all samples. When using the updated software, all fresh samples produced valid results, and only 0.2% of seeded samples produced invalid results. When substratified by ID probe, sensitivity for ID ranged from a high of 100% for S. marcescens to a low of 94.6% for CoNS. Indeterminant rates varied from 0 to 2.3%. The Accelerate Pheno system was designed to target common bloodstream pathogens (44–47), but coverage may vary depending on the local epidemiology and pathogen diversity of bloodstream infections. The organisms included in the Accelerate Pheno system are the organisms typically causing bloodstream infections with 65% Gram-positive organisms, 25% Gram-negative organisms, and 9.5% yeast. Since the FDA requires 300 specimens per drug (225 for drugs when testing organisms with a prevalence of less than 5%), for FDA clearance, the seeded challenge isolates were designed for on-panel targets, as is the standard.

An advantage of the Accelerate Pheno system is the monomicrobial call. The monomicrobial call is an attribute designed to provide laboratorians and clinicians with an indicator that the blood culture contains a single species; therefore, antimicrobial therapy could be reliably adjusted per Accelerate Pheno system AST results with a low risk of inappropriate antimicrobial de-escalation. Of fresh samples, 89% received a monomicrobial call. Note that the classification as “negative” for the monomicrobial call does not necessarily confirm the presence of multiple organisms. Use of the Gram stain, in conjunction with the monomicrobial call, yields a 99.4% PPV, i.e., only 1 in 100 positive results were in fact mixed. Therefore the risk of de-escalation under false pretenses is very low and should encourage physicians to follow antimicrobial stewardship guidelines for de-escalation when warranted.

Excellent concordance was obtained between the Accelerate Pheno system and the reference BMD method. Accurate detection of antimicrobial resistance resulting in prompt escalation of therapy is critical for a successful outcome when treating bacteremia. Studies have demonstrated that inappropriate empirical therapy is associated with increased hospital mortality (7, 9, 48). The need for rapid AST results has led to the development of several assays for ID, which cover 80 to 90% of pathogens recovered in positive blood cultures (12, 24, 49). However, unlike other rapid diagnostic platforms that identify organisms from positive blood culture bottles and detect genetic resistance markers, the Accelerate Pheno system is unique in its ability to identify and provide MIC and categorical phenotypic AST results in 7 h for several antimicrobial agents targeting the Gram-positive and Gram-negative organisms using the Accelerate PhenoTest BC kit. This is important because there is an association between high MICs within the susceptible range and adverse outcomes for patients with Gram-positive and Gram-negative bacterial infections. Regular surveillance of MICs is required due to a continuing decrease in susceptibility to the commonly used antibiotics in critically ill patients (50–52).

AST performance claims granted by FDA are limited by post-2007 guidelines that allow only clearance of organism/antimicrobial combinations listed in the clinical indications for use of the antimicrobial prescribing information. As a result, off-label combinations must be designated RUO, regardless of the assay performance. For Gram-positive organisms, the following organism/antimicrobial combinations were labeled RUO due to the absence of FDA breakpoints: doxycycline (Staphylococcus spp. and E. faecium), erythromycin (all coagulase-negative Staphylococcus spp.), TMP-SMX (Staphylococcus spp.), daptomycin (S. lugdunensis), and linezolid (all coagulase-negative Staphylococcus spp.), since these organism/antimicrobial combinations are not included in the FDA drug label. Furthermore, the ability of the Accelerate PhenoTest BC kit to detect resistance in the following combinations could not be determined because an insufficient number of resistant isolates were encountered at the time of comparative testing: ceftaroline and daptomycin (S. aureus); cefoxitin and MLSb for phenotypic resistance (S. lugdunensis) (see Table S5 in the supplemental material). Since daptomycin-nonsusceptible isolates were not encountered in this study, isolates yielding test results suggestive of a nonsusceptible category should be retested by a reference method. Due to the rare occurrence of such isolates, this is also a CLSI recommendation (53). Likewise, insufficient numbers of vancomycin-intermediate S. aureus (VISA) isolates were encountered such that the ability of the Accelerate PhenoTest BC kit to detect VISA is unknown.

Both resistance phenotype tests (MRSA/MRS and MLSb) showed excellent agreement (>96%) with all organisms tested (Table 4). The Accelerate Pheno system provides reductions in time to reporting MRSA/MSSA and vancomycin resistance in enterococcal bacteremia and also provides MIC data on therapeutic treatment options (e.g., daptomycin) 1 to 2 days sooner. The phenotypic expression of methicillin resistance can be variable in S. aureus. As such, an MIC result allows detection of non-*mecA*-mediated resistance mechanisms, such as *mecC*, hyperexpression of beta-lactamase (*blaZ*), or alterations to other penicillin-binding proteins (PBPs) that are often undetected by molecular methods. As a result, clinicians can gain earlier recognition of patients on suboptimal therapy and select the most likely patients to benefit from antibiotic escalation.

While the overall AST accuracy for Gram-positive bacteria was high, there were eight VMEs for the Gram-positive MIC and phenotypic susceptibilities (one each with the sole daptomycin-nonsusceptible S. aureus, CoNS and erythromycin, CoNS and MLSb, and five for CoNS and cefoxitin). Most of the MEs observed were with S. aureus and trimethoprim-sulfamethoxazole, S. lugdunensis and trimethoprim-sulfamethoxazole, and S. aureus and doxycycline (Table 5). While useful for de-escalation, these drugs are not first-line antibiotics for the treatment of Staphylococcus bloodstream infections. The ability of the Accelerate PhenoTest BC kit to test S. aureus with MLSb was not claimed, because performance was outside FDA acceptance criteria.

The Accelerate Pheno system received a *de novo* classification from the FDA because the technology is the only phenotypic AST system that performs testing directly from positive blood cultures. Several laboratories have resorted to using off-label direct blood culture susceptibilities on automated blood culture instruments, particularly for Gram-negative organisms (54–61). However, the direct AST methods are not standardized for such testing, varying substantially across laboratories, underscoring the need for an FDA-cleared system with well-documented performance specifications.

In the present study, the Accelerate Pheno system was rigorously compared to a CLSI BMD reference standard performed in a blind manner in triplicate. The results with the Accelerate Pheno system in this multicenter study are similar to the results of a study of the same platform from a single center in southern Germany and a second more recent paper by Brazelton de Cárdenas et al. from a pediatric hospital in the United States (60, 61). In the German study, of 115 episodes of Gram-negative bacteremia, the overall CA compared to the Vitek 2 system and Etest susceptibility results was 96.4%. Compared to the culture-based methods used for AST, time to result was reduced by 40.39 h (*P* < 0.0001). In that study, using analysis software version 1.1.0.69, however, the VME and ME rates (1.0% and 2.3%, respectively) were higher than what was observed in our study (0.5% and 0.9%, respectively). In the Brazelton de Cárdenas study, performed at a pediatric oncology center using 104 specimens, the overall CA ranged from 91.2 to 91.8% compared to the Vitek 2 compact (AST-GN69) and BMD depending upon the interpretive guidelines used (FDA versus CLSI) (61). There were no VMEs using BMD as the comparator, 1.4% MEs and 7.4% mEs for the Gram-negative rods tested. The mean times for susceptibility results were 46.7 h for the Vitek 2 and 6.6 h for the Accelerate Pheno system.

For Gram-negative bacteria, there were eight VMEs (0.5%) observed in our study, half of which occurred with colistin when testing A. baumannii (*n* = 1), E. coli (*n* = 1), and E. cloacae (*n* = 2) complex (using EUCAST breakpoints for the Enterobacteriaceae). The results with colistin are more favorable with the Accelerate Pheno system than what has been published for other automated systems. In the study by Vourli et al., compared to BMD, both the BD Phoenix 100 system (NMIC/ID-96 panel) and the Vitek 2 compact (AST XN05 card) greatly underestimated colistin resistance (41.4% and 37.9% VME, respectively) among 117 carbapenem-resistant A. baumannii (CRAB) isolates (62). In the more limited study by Dafopoulou et al., the authors compared six susceptibility testing methods on 61 carbapenem-resistant K. pneumoniae isolates and 20 A. baumannii isolates. In that study, Vitek 2 (AST-EXN8 card) produced no VMEs, but Etest and an MIC test strip had unacceptably high VMEs for colistin (63).

In our study, there were a total of 43 MEs (0.9%) for Gram-negative bacteria. Most of these fell into two patterns in that nearly a third (*n* = 14; 32.6%) were observed when testing colistin and various organisms (A. baumannii, E. coli, and E. cloacae complex) and 12 errors occurred with P. aeruginosa, 9 of which occurred when testing ceftazidime (*n* = 6) and cefepime (*n* = 3). However, there are no intermediate breakpoints for these three drugs. These MEs resulted in a lowering of the CA for ceftazidime below 90%, and resistance to both agents should be confirmed. Laboratories may consider performing off-label verification using CLSI breakpoints for these antimicrobials and P. aeruginosa, as retesting organisms by another method is time-consuming and, more importantly, causes delays in targeted therapy.

Nonetheless, when using the FDA breakpoints and taking into consideration the 11 mEs for piperacillin-tazobactam, the Accelerate Pheno system is less reliable when testing P. aeruginosa compared to the CLSI reference BMD method. This is not unique to the Accelerate Pheno system in that Vitek 2 requires performance of an alternative method of testing when a resistant result is obtained for piperacillin-tazobactam with P. aeruginosa. Clearly, more data are needed when testing P. aeruginosa (especially resistant strains) with the Accelerate Pheno system and any other commercial method.

Consistent with published comparative series of broad patient populations, A. baumannii was an infrequent cause of bacteremia (*n* = 3 in the present study; range in literature of 0 to 1.7% of all Gram-negative bacteremias) (54, 55, 57–59). However, outside the United States, and among certain patient populations such as cancer patients, burn patients, and ICU patients, this organism is increasing in frequency, as is its resistance (64, 65). Importantly, testing all 93 meropenem-resistant A. baumannii isolates revealed no categorical errors. Having a rapid, reliable method for testing A. baumannii as seen in this study to RUO agents meropenem, colistin, and minocycline for carbapenem-resistant strains would be an asset when treating one of the most challenging pathogens encountered in some settings (66). Currently, laboratories are limited in their ability to test this organism using commercial systems, and there are almost no FDA breakpoints for this organism (66).

In the present study, ampicillin-sulbactam had low CA due to a large number of mEs, but piperacillin-tazobactam performed well against A. baumannii. The results for minocycline look promising with EA and CA greater than 92% and no VMEs. These results are similar to those recently reported by Wang et al. when testing 107 CRAB isolates against the tetracyclines using Etest, disk diffusion, and Sensititre BMD methods (67). VMEs (2.8%) were higher than in the present study (67). MEs were high with the Etest method, and mE rates for minocycline were above 14% for Etest and disk diffusion (67), similar to the 17% in the present study.

When the performance of the Accelerate Pheno system for testing Enterobacteriaceae is compared to the literature, the EA and CA are very similar to what has been reported (54, 68), with the exception of cefazolin. Using CLSI breakpoints, there were 39 mEs with cefazolin thus lowering the CA to 85.8%; however, 26 of these were within EA. Cefazolin was not submitted for FDA clearance and therefore remains RUO. This is a problem for other commercial systems as well, because the cefazolin-susceptible breakpoint bisects the wild-type distribution; as a result, the expected 1-dilution variability of MIC testing yields CA errors. Minor errors were also seen with amikacin (4.9%) and tobramycin (4.0%), but not with gentamicin, and these rates are slightly higher than reported in other comparison studies of other automated systems (56, 57, 59, 68), but still below the 10% acceptable rate of the FDA. High rates of mEs for the aminoglycosides among automated systems have been reported in some studies, specifically when testing gentamicin-resistant and carbapenem-resistant Enterobacteriaceae (69, 70). When testing the ertapenem and meropenem carbapenems, there were no VMEs and five MEs were seen among Enterobacter spp. (two for ertapenem, three for meropenem). For the Accelerate Pheno system, there were high mE rates among the β-lactam/β-lactamase inhibitor combinations (15.2% for ampicillin-sulbactam and 6% for piperacillin-tazobactam) (Table 6). These rates are higher for ampicillin-sulbactam and comparable to the rates reported for piperacillin-tazobactam in the survey of the Vitek 2 using manufacturer's breakpoints by Bobenchik et al. (0 to 8% for ampicillin-sulbactam and 5 to 14.4% for piperacillin-tazobactam among various Enterobacteriaceae species) (8% mE for Klebsiella sp., E. coli, and P. mirabilis combined) (68). Minor error rates are higher for both drugs compared to those reported in the studies of direct testing of bacteremia isolates (54, 56). In the study by Marschal et al. of the Accelerate Pheno system compared to culture-based AST, there were no mEs, but the ME rates were 8.8% for ampicillin-sulbactam (Enterobacteriaceae only) and 8.2% for piperacillin-tazobactam (Enterobacteriaceae and P. aeruginosa combined), emphasizing the variability among comparative reference methods (60).

As indicated in Table S5 in the supplemental material, this study has limitations in that drug resistance was encountered infrequently during the study period and only with a few organisms (Fig. 1) (14). Despite the limitations, the advantages of the Accelerate Pheno system included a much more rapid evaluation of positive blood cultures than other phenotypic susceptibility systems. An additional advantage is the Accelerate Pheno system avoids the need for an isolate to obtain antibiotic susceptibilities, if additional testing is not required. However, it is likely that samples will continue to be subcultured for archiving isolates. A cultivated isolate would also be necessary if further work up is needed, e.g., for confirmation of unusual susceptibility or for epidemiological purposes and when results are needed for antimicrobial agents not included on the panel. In contrast, molecular tests still require AST for at the very least Gram-negative bacteria, since rapid PCR-based platforms target a limited number of organisms and resistance genes. Importantly, the Accelerate Pheno system can detect phenotypic resistance of organisms to a number of antibiotics regardless of mechanism (for example, AmpC, porin alterations, and efflux pumps), that is, phenotypic resistance that is not detected by current commercial molecular methods. Likewise, potentially novel methods of resistance introduced into a population can also be detected. This is especially valuable for Gram-negative organisms that can have many mechanisms of resistance that are difficult to encompass in a single molecular test. The phenotypic susceptibilities offer additional value for rapid bacteremia/sepsis patient intervention, e.g., Gram-negative infections, MIC-based pharmacokinetic (PK)/pharmacodynamic (PD) antibiotic dosing, and when a pathogen expresses a resistance mechanism that is not included in the molecular test panel, or any available panel for that matter.

Providing appropriate empirical coverage is proving more and more difficult as antibiotic resistance increases in both the hospital and the community. Hospitals that have established stewardship programs around rapid pathogen ID and resistance mechanism detection methods directly from positive blood cultures substantially reduce the time to clinically actionable results (71, 72). Unfortunately, many hospitals do not have access to physicians and/or pharmacists with infectious disease training to interpret molecular results that provide pathogen ID and resistance mechanisms. For institutions that are building their stewardship program and for hospitals that have established stewardship, rapid pathogen ID in 90 min, determination of MRSA/MSSA and vancomycin-resistant enterococcus (VRE), and determining phenotypic susceptibilities to multiple antibiotics for Gram-positive and Gram-negative organisms 1 to 2 days sooner than current methods can provide useful information. Results can be integrated into electronic decision support and provided in the “Susceptible, Intermediate, and Resistant” format with templated comments to provide actionable suggestions for general practitioners. It is conceivable that susceptible results from the Accelerate Pheno system may lead to rapid de-escalation of broad-spectrum antibiotics even without an active pharmacist/stewardship intervention. The Accelerate Pheno system may prove to be a valuable tool which could prove significant in therapeutic management of bacteremia, the leading cause of sepsis. Further clinical trial outcome studies are required to establish the impact on patient care.

## Supplementary Material

Supplemental material
