# Supplementary material for: Multicenter Evaluation of the Accelerate PhenoTest BC Kit for Rapid Identification and Phenotypic Antimicrobial Susceptibility Testing Using Morphokinetic Cellular Analysis
Source: J Clin Microbiol. 2018 Mar 26;56(4):e01329-17. doi: 10.1128/JCM.01329-17 (PMC5869823; doi:10.1128/JCM.01329-17)
Supplement: Supplemental material [file supp_56_4_e01329-17__index.html]

Multicenter Evaluation of the Accelerate PhenoTest BC Kit for Rapid Identification and Phenotypic Antimicrobial Susceptibility Testing Using Morphokinetic Cellular Analysis — Supplemental material 

# Multicenter Evaluation of the Accelerate PhenoTest BC Kit for Rapid Identification and Phenotypic Antimicrobial Susceptibility Testing Using Morphokinetic Cellular Analysis

## Supplemental material

- Supplemental file 1 -

  Supplemental methods; Fig. S1 (Sample flowchart of specimen disposition at time of FDA clearance); and Tables S1 (Gram-positive target organism/antimicrobial combinations with reportable ranges), S2 (Gram-negative target organism/antimicrobial combinations with reportable ranges), S3 (Identification performance by DNA probe at FDA clearance), S4 (Invalid, indeterminate, and false-positive rates by sample type), and S5 (Accelerate PhenoTest BC kit limitations)

  PDF, 385K
