# Supplementary material for: Multicenter Evaluation of the Accelerate PhenoTest BC Kit for Rapid Identification and Phenotypic Antimicrobial Susceptibility Testing Using Morphokinetic Cellular Analysis
Source: J Clin Microbiol. 2018 Mar 26;56(4):e01329-17. doi: 10.1128/JCM.01329-17 (PMC5869823; doi:10.1128/JCM.01329-17)
Supplement: Supplemental material [file JCM.01329-17_zjm999095863s1.pdf]

Supplemental Materials

Definitions/Abbreviations

Categorical Agreement (CA) – Percent of all test results with same category result as the reference result.

Essential Agreement (EA) – Percent of all test results within one 2-fold dilution of the reference result

False negative (FN) – Test negative and reference positive

False positive (FP) – Test positive and reference negative

Gram stain resolved/mitigated – Accelerate Pheno system incorrect identification result that could be arbitrated by comparison with Gram stain results. (i.e. Accelerate Pheno™ system *Staphylococcus aureus* identification result determined to be a false-positive due to comparison with a Gram stain result containing only Gram-negative rods.)

Gram stain unresolved/unmitigated - Accelerate Pheno system incorrect identification result that could not be arbitrated by comparison with Gram stain results. (i.e. Accelerate Pheno™ system *Klebsiella* spp. identification result that was actually *E. coli*. Comparison with a Gram stain result containing only Gram-negative rods would fail to resolve.)

Invalid – No results reported for a sample

Indeterminate – No results reported for a FISH ID probe

Major Error (ME) – Percent of reference-susceptible results that are test resistant (false-resistance)

Minor Error (mE) – Percent of all test results where one result (test or reference) is intermediate and the other is not.

Negative Percent Agreement (NPA) – The number of true negative results for a diagnostic test divided by the number of negative results by the reference method. Same calculation as specificity.

Positive Percent Agreement (PPA) – The number of true positive results for a diagnostic test divided by the number of positive results by the reference method. Same calculation as sensitivity.

Positive Predictive Value (PPV) – The number of true positive results divided by all positive results for a diagnostic test.

Sensitivity (clinical) – How often a diagnostic test is positive in diseased patients

Specificity (clinical) – How often a diagnostic test is negative in non-diseased patients

True negative (TN) = Test negative and reference negative

True positive (TP) = Test positive and reference positive

Valid – Results reported for a sample

Very Major Error (VME) – Percent of reference-resistant results that are test susceptible (false-susceptible)

Equations

Essential Agreement Rate: %EA = [100x(Number of test MIC results within one doubling dilution of reference MIC)]/(Total number of results)

Categorical Agreement Rate: %CA = [100x(Number of test results in categorical agreement with reference)]/(Total number of results)

Indeterminate Rate: %IND = (100xIND)/(total tests)

Invalid Rate: %INV = (100xINV)/(total tests)

Major Error Rate: %ME = [100x(Number of false-resistant test results)]/(Total susceptible results by reference)

Minor Error Rate: %mE = [100x(Number of results where one result is intermediate and the other result is susceptible or resistant)]/(Total number of results)

Positive Predictive Value: PPV = (100xTP)/(TP+FP)]

Sensitivity/PPA = (100xTP)/(TP+FN)

Specificity/NPA = (100xTN)/(TN+FP)

Very Major Error Rate: %VME = [100x(Number of false-susceptible test results)]/(Total resistant results by reference)

Methods

Study Phases. In Phase I, fresh BC samples were tested and analyzed at 11 of the 13 clinical sites participating in the study, and seeded samples were provided, tested, and analyzed at 11 sites. Nine clinical sites tested both fresh and seeded samples. In Phase II, five of the clinical sites enrolled and tested positive BC prepared from fresh and seeded samples with the Accelerate Pheno™ system. Reference testing was performed at the reference laboratory Phase

III was carried out at Accelerate Diagnostics, Inc. (Tucson, AZ) using only seeded samples and all reference testing was performed on-site during this phase. Phases II and III provided additional data for certain organism/antimicrobial combinations for which there was insufficient enrollment in Phase I.

Bottle Types. BACTEC™ (n=10) (Becton Dickinson, Baltimore, MD), BacT/ALERT® (n=1) (bioMérieux, Marcy-l'Étoile, France), or VersaTREK® (n=1) (Thermo Fisher Scientific, Waltham, MA) blood culture systems were used at the clinical sites. Accelerate Diagnostics, Inc. used both BACTEC™ and VersaTREK® bottles. BacT/ALERT® SA Standard Aerobic and SN Anaerobic bottles, BACTEC™ Standard/10 Aerobic/F Medium, BACTEC™ Standard Anaerobic/F Medium, BACTEC™ Plus Aerobic/F Medium, BACTEC™ Plus Anaerobic/F Medium, BACTEC™ PEDS PLUS/F Medium, BACTEC™ Lytic/10 Anaerobic/F Medium, VersaTREK® Redox 1 Aerobic Media, and VersaTREK® Redox 2 Anaerobic Media were used with their corresponding systems. Fresh samples from mycobacterial type blood culture media (BACTEC™ Myco/F Lytic, BacT/ALERT® MP Bottle, VersaTREK® Myco) were excluded.

Seeded Sample Preparation. Seeded samples were prepared by spiking clinical isolates into blood culture media containing 10 mL of commercially available healthy human whole blood (Bioreclamation, Baltimore, MD) collected in 6% w/v sodium polyanetholesulfonate (SPS; Sigma Aldrich, St. Louis, MO). Seeded bottles were loaded onto automated blood culture instruments and incubated according to the system's specifications. Organisms for spiking were suspended in normal saline and standardized to a 0.5 McFarland concentration for bacteria (~1.5 x 10^8 CFU/mL), or a 2 McFarland concentration for yeast (~5 x 10^6 CFU/mL). The suspensions underwent three 1:100 serial dilutions before inoculating the blood culture bottle with 500 µL to produce a final concentration of ~75 CFU per bottle.

Frozen Isolate Preparation at Clinical Site for AST Discrepancy Testing. Frozen isolates were prepared from sub-cultured plates as described earlier. The plates used for preparation of frozen isolates were sub-cultured from refrigerated sub-culture plates of the original positive blood culture. In some cases, the sub-cultured plates of the original positive blood culture were removed from the refrigerator, parafilmed and sent to Accelerate Diagnostics, Inc. directly.

Reference AST Testing. The reference standard for AST comparator testing were the Clinical and Laboratory Standard Institute (CLSI) reference frozen broth microdilution (BMD) panels made in-house at Accelerate Diagnostics, Inc., and shipped to the reference laboratory. BMD panels were prepared using cation-adjusted Mueller Hinton Broth (CAMHB) (BD, Difco, Catalogue number 275730) and antibiotics obtained from Merck, Sigma, USP and Pfizer. Cation concentrations for CAMHB were tested externally using ICP-OES as required by CLSI, and every BMD lot underwent biological quality control testing per CLSI standards (1, 2). In order to account for MIC variability seen in BMD for some isolates, each isolate was tested in triplicate (1) and the modal MIC was used as the comparator. If no modal MIC was obtained, triplicate BMD was repeated for the sample and the modal MIC was used from the results of the six repeats. If no modal MIC was obtained after 6 replicate BMD tests, the sample was excluded from analysis. For cefoxitin testing of staphylococci, disk diffusion was performed with FOX-30 disks (Hardy Diagnostics, Santa Maria, CA, Catalog number 231590) on MHA plates (Becton Dickinson). For cases in which the test failed (due to sparse growth, hazy or double zones, heterogeneous growth) or if the disk diffusion zone of growth inhibition was within 1 mm of the breakpoint, testing was repeated in triplicate and the modal category from the 4 repeats used as

the comparator. If no modal category was obtained after repeat testing, the sample was excluded from analysis.

Quality Control (QC) Testing on the Accelerate Pheno™ system. For the Accelerate Pheno™ system, one QC panel (EPEES, SES, CASKS, Table 1) was run on one module per day. The panels were rotated such that each panel was tested at least once per week. QC testing was rotated between modules such that all modules were used to perform QC at approximately the same frequency. QC panels were run per manufacturer instructions (3).

Table 1: QC panels and organisms for ID and/or AST

QC Panel	Species	Strain Number
EPEES (AST)	*Escherichia coli*	ATCC® 25922™
	Escherichia coli	ATCC® 35218™
	Pseudomonas aeruginosa	ATCC® 27853™
	Enterococcus faecalis	ATCC® 29212™
	Staphylococcus aureus	ATCC® 29213™
SES (AST)	*Enterococcus faecalis*	ATCC® 51299™
	Staphylococcus aureus	ATCC® 43300™
	Staphylococcus aureus	ATCC® BAA-977™
CASKS (ID)	*Staphylococcus lugdunensis*	ATCC® 700328™
	Klebsiella pneumoniae	ATCC® 700603™
	Acinetobacter baumannii	ATCC® 19606™
	Citrobacter freundii	ATCC® 6879™
	Streptococcus agalactiae	ATCC® 12403™
	Candida glabrata	ATCC® 2001™

References

1. CLSI. Methods for Dilution Antimicrobial Susceptibility Tests for Bacteria That Grow Aerobically; Approved Standard—Ninth Edition. CLSI document M07-A9 (ISBN 1-56238-783-9 [Print]; ISBN 1-56238-784-7 [Electronic]). Clinical and Laboratory Standards Institute. , 950 West Valley Road, Suite 2500, Wayne, Pennsylvania 19087, USA, 2012.
2. CLSI. 2016. Performance Standards for Antimicrobial Susceptibility Testing. 26th ed. CLSI supplement M100S (ISBN 1-56238-923-8 [Print]; ISBN 1-56238-924-6 [Electronic]). Clinical and Laboratory Standards Institute, 950 West Valley Road, Suite 2500, Wayne, Pennsylvania 19087, USA.
3. Diagnostics A. 2017. Accelerate PhenoTest™ BC kit Instructions for Use.

FIG S1 Sample Flowchart of Specimen Disposition at Time of FDA Clearance

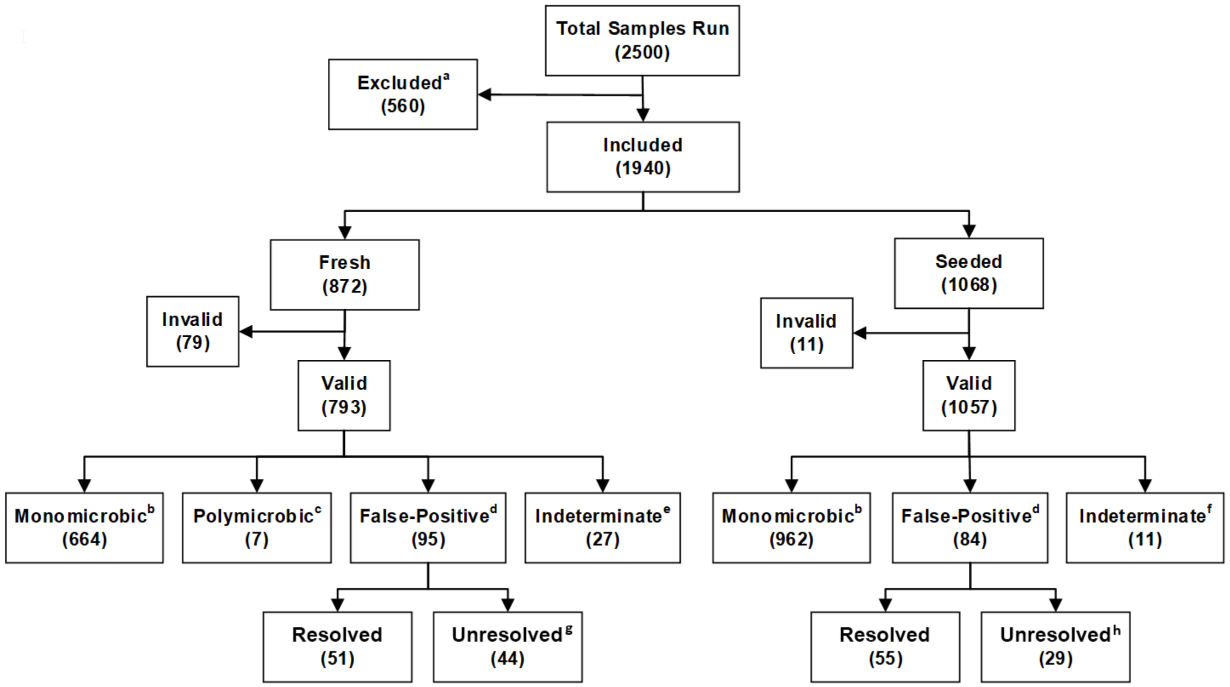

[a]Protocol Deviation (n=216); Experiments Halted (n=26); Experiments Never Ran (n=15); Bottle >8 h Post-Positivity (n=31); Gram stain shows no organism (n=24); Isolate Not Received at Reference Laboratory (n=18); Isolate Received Later than 4 days from Media Preparation (n=3); ID Reference Growth Failure (n=29); Non-Pure Isolate (n=169); ID Reference Purity Plate Failure (n=6); Invalid ID Reference Result (n=1); Accelerate Pheno™ System Run State Not "Complete" (n=22)

[b]Single on-panel organism reported

[c]Polymicrobial samples where Accelerate PhenoTest™ BC kit ID results exactly match the reference

[d]Monomicrobial or polymicrobial runs containing any false positive(s)

[e]Fresh samples with only indeterminate/negative results = 27, Fresh samples including indeterminate result(s) = 74

[f]Seeded samples with only indeterminate/negative results = 11, Seeded samples including indeterminate result(s) = 198

[g]Of the 44 fresh unresolved false-positives, 38 showed genus-level agreement, while the remaining six were one *E. coli* called *E. coli*+CNS+*Enterobacter* spp., one *S. aureus*+*Pantoea* spp. mix called *Klebsiella* spp., one *Klebsiella pneumoniae* called *Klebsiella* spp.+*E. coli*, one *Lactococcus raffinolactis* called *Streptococcus* spp., one *Streptococcus parasanguinis* called *Streptococcus* spp.+*C. glabrata* and one *Acinetobacter lwoffii* called *A. baumannii*+*Enterobacter* spp. by the Accelerate Pheno™ system.

[h]Of the 29 seeded samples containing unresolved false-positives, 16 showed genus-level agreement, while the remaining 13 were one *Enterococcus cecorum* called *E. faecalis*+*C. glabrata*, one *Pantoea* spp. called *Enterobacter* spp., one *C. koseri* called *Citrobacter* spp.+*Proteus* spp., two *C. koseri* called *Citrobacter* spp.+*Klebsiella* spp., one *C. freundii* called *Citrobacter* spp.+*P. aeruginosa*, one *C. koseri* sample called *Citrobacter* spp.+*E. faecium*+*Klebsiella* spp., one *E. cloacae* complex and one *E. aerogenes* called *Enterobacter* spp.+*P. aeruginosa*, one *P. mirabilis* called *Proteus* spp.+*E. coli*, one *E. coli* called *E. coli*+*Citrobacter* spp., one *Streptococcus pyogenes* called *Streptococcus* spp.+*E. faecium*, and one *S. marcescens* called *S. marcescens*+*A. baumannii*+*Enterobacter* spp.+*P. aeruginosa*+*S. aureus* by the Accelerate Pheno™ system.

Table S1: Gram-positive Target Organism/Antimicrobial Combinations with Reportable Ranges

Class	Antimicrobial[a]	ID Target				
		SAU	SLU	CNS	EFM	EFS
Penicillins	Ampicillin				2-32	2-32
Cephalosporins	Ceftaroline	0.25-8				
Lipopeptides	Daptomycin	0.25-2	0.25-2[b]	0.25-2	1-8	1-8
Tetracyclines	Doxycycline[b]	1-32	1-32	1-32	2-32	
Macrolides	Erythromycin	0.12-16	0.12-16[b]	0.12-16[b]		
Oxazolidinones	Linezolid	1-16	1-16[b]	1-16[b]	0.5-16	0.5-16
Folate pathway inhibitors	Trimethoprim-Sulfamethoxazole[b]	0.5-8	0.5-8			
Glycopeptides	Vancomycin	0.5-32	1-64	1-64	1-64	1-64
Resistance Phenotypes	Cefoxitin (MRSA)	Pos/Neg	Pos/Neg	Pos/Neg		
	Erythromycin-Clindamycin (MLSb)		Pos/Neg	Pos/Neg		

Abbreviations: SAU=*Staphylococcus aureus*; SLU=*Staphylococcus lugdunensis*; CNS=Coagulase-negative *Staphylococcus* spp. (i.e., *Staphylococcus epidermidis*, *Staphylococcus haemolyticus*, *Staphylococcus hominis, Staphylococcus capitis, Staphylococcus lugdunensis, Staphylococcus warneri,* not differentiated); EFM=*Enterococcus faecium*; EFS=*Enterococcus faecalis*; STR=*Streptococcus* spp. (i.e., *Streptococcus mitis, Streptococcus oralis, Streptococcus gallolyticus, Streptococcus agalactiae, Streptococcus pneumoniae*, not differentiated)

[a]Antimicrobial concentrations are listed in µg/mL.

[b]RUO

Table S2: Gram-negative Target Organism/Antimicrobial Combinations with Reportable Ranges

		ID Target							
**Class**	**Antimicrobial[a]**	**ECO**	**KLE**	**ENT**	**CIT**	**PRO**	**SMA**	**PAE**	**ABA**
Aminoglycosides	Amikacin	4-128	4-128	4-128	4-128	4-128	4-128	4-128	4-128
	Gentamicin	1-32	1-32	1-32	1-32	1-32	1-32	1-32	
	Tobramycin	1-32	1-32	1-32	1-32	1-32	1-32	1-32	
Carbapenems	Ertapenem	0.12-4	0.12-4	0.12-4	0.12-4	0.12-4	0.12-4		
	Meropenem	0.25-8	0.25-8	0.5-8	0.25-8	0.25-8	0.25-8	1-16	0.5-16[b]
Cephalosporins	Cefazolin[b]	0.5-16	0.5-16						
	Cefepime	1-32	1-32	1-32	1-32	1-32	1-32	2-32	2-64[b]
	Ceftazidime	2-32	2-32	2-32	2-32	1-32	1-32	2-32	
	Ceftriaxone	0.25-8	0.25-8	0.25-8	0.25-8	0.5-8	0.5-8		
Fluoroquinolones	Ciprofloxacin	0.25-8	0.25-8	0.5-8	0.25-8	0.25-8	0.25-8	0.25-8	0.25-8[b]
Monobactams	Aztreonam	1-32	1-32	1-32	1-32	1-32	1-32		
BL-BLIs	Ampicillin-Sulbactam	2-64	2-64			4-64			2-64[b]
	Piperacillin-Tazobactam	4-256	4-256	4-256	4-256	4-256	4-256	8-256	4-256
Polymyxins	Colistin[b]	0.5-8	0.5-8	0.5-8	0.5-8			0.5-16	0.5-8
Tetracyclines	Minocycline[b]								1-32

Abbreviations: BL-BLIs=Beta-lactam / beta-lactamase inhibitors; ECO=*Escherichia coli*; KLE=*Klebsiella* spp. (i.e., *Klebsiella pneumoniae, Klebsiella oxytoca*, not differentiated); ENT=*Enterobacter* spp. (i.e., *Enterobacter cloacae, Enterobacter aerogenes*, not differentiated); CIT=*Citrobacter* spp. (i.e., *Citrobacter freundii*, *Citrobacter koseri*, not differentiated); PRO=*Proteus* spp. (i.e., *Proteus mirabilis*, *Proteus vulgaris*, not differentiated); SMA=*Serratia marcescens*; PAE=*Pseudomonas aeruginosa*; ABA=*Acinetobacter baumannii*

[a]Antimicrobial concentrations are listed in µg/mL.

[b]RUO

TABLE S3 Identification Performance by DNA Probe at FDA Clearance

					Sensitivity		Specificity	
**Probe**	**TP**	**FN**	**TN**	**FP**	**%**	**95% CI**	**%**	**95% CI**
**Gram-Positive**								
Staphylococcus aureus	238	5	1548	24	97.9	(95.3-99.1)	98.5	(91.1-98.3)
CNS[a]	243	12	1458	27	95.3	(92.0-97.3)	98.2	(97.4-98.8)
Staphylococcus lugdunensis	77	2	1748	1	97.5	(91.2-99.3)	99.9	(99.7-100.0)
Enterococcus faecium	100	2	1724	15	98	(93.1-99.5)	99.1	(98.6-99.5)
Enterococcus faecalis	98	3	1726	2	97	(91.6-99.0)	99.9	(99.6-100)
Streptococcus spp.	171	5	1615	39	97.2	(93.5-98.)	97.6	(96.8-98.3)
Gram-Positive Total	927	29	9819	108	97	(95.7-97.9)	98.9	(98.7-99.1)
**Gram-Negative**								
Escherichia coli	92	3	1677	12	96.8	(93.3-98.9)	99.3	(99.3-99.9)
Klebsiella spp.	144	4	1690	5	97.3	(91.1-98.3)	99.7	(99.2-99.8)
Enterobacter spp.	122	5	1651	6	96.1	(92.3-99.1)	99.6	(99.0-99.7)
Proteus spp.	107	3	1682	9	97.3	(92.1-99.4)	99.5	(99.2-99.8)
Citrobacter spp.	86	2	1751	7	97.7	(91.1-98.9)	99.6	(98.8-99.6)
Serratia marcescens	51	0	1795	2	100	(93.0-100.0)	99.9	(99.6-100)
Pseudomonas aeruginosa	58	0	1779	10	100	(93.8-100)	99.4	(99.0-99.7)
Acinetobacter baumannii	68	1	1764	6	98.6	(92.2-99.9)	99.7	(99.3-99.8)
Gram-Negative Total	728	18	13789	57	97.6	(96.2-98.5)	99.6	(99.5-99.7)
**Yeast**								
Candida albicans	45	0	1777	8	100	(92.1-100)	99.6	(99.1-99.8)
Candida glabrata	50	0	1754	29	100	(92.9-100)	98.4	(97.7-98.87)
Yeast Total	95	0	3531	37	100	(96.1-100)	99	(98.6-99.3)
**Overall**	1750	47	27139	202	97.4	(96.5-98.0)	99.3	(99.2-99.4)

[a]Coagulase-Negative *Staphylococcus* spp.

TABLE S4 Invalid, Indeterminate and False Positive Rates by Sample Type

	FDA Clearance			2017 Software Update		
	**Fresh**	**Seeded**	**Overall**	**Fresh**	**Seeded**	**Overall**
N. Total Samples	872	1068	**1940**	872	1068	**1940**
Invalid	79 (9.1%)	11 (1.0%)	**90 (4.6%)**	0 (0.0%)	2 (0.2%)	**2 (0.1%)**
Valid	793 (90.9%)	1057 (99.0%)	**1850 (95.4%)**	872 (100%)	1066 (99.8%)	**1938 (99.9%)**
Indeterminate[a,b]	74 (9.3%)	198 (18.7)	**272 (14.7%)**	39 (4.5%)	6 (0.6%)	**45 (2.3%)**
False Positives[b]	95 (12.0%)	84 (7.9%)	**179 (9.7%)**	83 (9.5%)	60 (5.6%)	**143 (7.4%)**
Resolved False Positives[c]	51 (53.7%)	55 (65.5%)	**106 (59.2%)**	35 (42.2%)	36 (60.0%)	**71 (49.7%)**
Unresolved False Positives[c]	44 (46.3%)	29 (34.5%)	**73 (40.8%)**	48 (57.8%)	24 (40.0%)	**72 (50.3%)**

[a]Samples with any indeterminate results.
[b]Rates calculated out of total valid results
[c]Rates calculated out of total false positives

Table S5: Accelerate PhenoTest™ BC Kit Limitations

Category	Limitation Language	Antibiotic	Bacteria
Insufficient number of R strains	The ability of the Accelerate PhenoTest™ BC kit to detect resistance in the following combinations is unknown because an insufficient number of resistant isolates were encountered at the time of comparable testing:	Amikacin	*Citrobacter* spp., *Enterobacter* spp., *E. coli, Proteus* spp., *S. marcescens*
		Aztreonam	*Proteus* spp., *S. marcescens*
		Cefepime	*Citrobacter* spp., *Proteus* spp., *S. marcescens*
		Ceftazidime	*Proteus* spp., *S. marcescens*
		Ceftaroline	*S. aureus*
		Ceftriaxone	*Citrobacter* spp., *E. cloacae, S. marcescens*
		Ciprofloxacin	*Citrobacter* spp., *Proteus* spp., *S. marcescens*
		Daptomycin	*S. aureus*
		Ertapenem	*Citrobacter* spp., *Proteus* spp., *and S. marcescens*
		Gentamicin	*Citrobacter* spp., *Enterobacter* spp., *Proteus* spp., *S. marcescens*
		Meropenem	*Citrobacter* spp., *E. coli, Proteus* spp., and *S. marcescens*
		Piperacillin/Tazobactam	*Proteus* spp., and *S. marcescens*
		Tobramycin	*Citrobacter* spp., *Proteus* spp., *S. marcescens*
		Cefoxitin (Methicillin Resistance)	*S.lugdunensis*
		Erythromycin/Clindamycin (MLSb)	*S.lugdunensis*
Insufficient number of NS strains	The current absence of data on daptomycin-resistant isolates precludes defining any categories other than "susceptible". Isolates yielding test results suggestive of a non-susceptible category should be retested and if the result is confirmed, the isolate should be retested using the reference method.	Daptomycin	*Staphylococcus* spp. (excluding *S. lugdunensis*), *Enterococcus* spp.
Insufficient number of VISA strains	The ability of the Accelerate PhenoTest™ BC kit to detect vancomycin-intermediate *Staphylococcus aureus* isolates (VISA) is unknown because insufficient numbers of VISA isolates were evaluated at the time of comparative testing.	Vancomycin	*S. aureus*

Category	Limitation Language	Antibiotic	Bacteria
Major error limitations	The following antimicrobial/organism combinations may produce a resistant result that can be found susceptible by the reference method. If critical to patient care confirm these results with an alternate method:	Ceftazidime	*P. aeruginosa* (Any *P. aeruginosa* isolate that provides an MIC ≥16 µg/mL should be retested using an alternate method)
		Cefepime	*P. aeruginosa*
		Ertapenem	*Enterobacter* spp.
		Meropenem	*Enterobacter* spp.
		Piperacillin/Tazobactam	*A. baumannii*
		Piperacillin/Tazobactam	*Klebsiella* spp.
Essential agreement limitation	Due to a low essential agreement for *Serratia marcescens* with ceftriaxone, results should be confirmed with an alternate method if critical to patient care.	Ceftriaxone	*S. marcescens*
Inaccurate MIC's in the R range	The ability of the Accelerate PhenoTest™ BC kit to provide accurate MICs with amikacin resistant strains of *A. baumannii* has not been established; isolates of this species that provide resistant results should be confirmed by an alternative method.	Amikacin	*A. baumannii*
MIC's tend to be one dilution higher than reference	Accelerate PhenoTest™ BC kit "antibiotic" MIC values for "organism" tended to be one doubling dilution higher than the reference MIC value.	Ampicillin/Sulbactam	Enterobacteraiaceae
		Aztreonam	
		Ceftazidime	
		Ertapenem	
MIC's tend to be one dilution lower than reference	Accelerate PhenoTest™ BC kit ceftazidime MIC values for *P. aeruginosa* tended to be one doubling dilution lower than the reference MIC value.	Ceftazidime	*P. aeruginosa*
